# EWI‐2 controls nucleocytoplasmic shuttling of EGFR signaling molecules and miRNA sorting in exosomes to inhibit prostate cancer cell metastasis

**DOI:** 10.1002/1878-0261.12930

**Published:** 2021-03-27

**Authors:** Chenying Fu, Qing Zhang, Ani Wang, Songpeng Yang, Yangfu Jiang, Lin Bai, Quan Wei

**Affiliations:** ^1^ State Key Laboratory of Biotherapy and National Clinical Research Center for Geriatrics West China Hospital Sichuan University Chengdu, Sichuan China; ^2^ Department of Rehabilitation Medicine Center Key Laboratory of Rehabilitation Medicine in Sichuan Province West China Hospital Sichuan University Chengdu China; ^3^ Cadiovascular Center The Fifth Affiliated Hospital Sun Yat‐sen University Zhuhai China; ^4^ Research Core Facility West China Hospital Sichuan University Chengdu China

**Keywords:** cancer metastasis, EGF receptor, EWI‐2/PGRL, exosomes, miR‐3934‐5p, nuclear translocation

## Abstract

Early and accurate diagnosis of prostate cancer (PCa) is extremely important, as metastatic PCa remains hard to treat. EWI‐2, a member of the Ig protein subfamily, is known to inhibit PCa cell migration. In this study, we found that EWI‐2 localized on both the cell membrane and exosomes regulates the distribution of miR‐3934‐5p between cells and exosomes. Interestingly, we observed that EWI‐2 is localized not only on the plasma membrane but also on the nuclear envelope (nuclear membrane), where it regulates the nuclear translocation of signaling molecules and miRNA. Collectively, these functions of EWI‐2 found in lipid bilayers appear to regulate PCa cell metastasis through the epidermal growth factor receptor‐mitogen‐activated protein kinase‐extracellular‐signal‐regulated kinase (EGFR‐MAPK‐ERK) pathway. Our research provides new insights into the molecular function of EWI‐2 on PCa metastasis, and highlights EWI‐2 as a potential PCa biomarker.

## Graphical abstract

Our findings identify EWI‐2 as a potential prostate cancer (PCa) biomarker. EWI‐2 is involved in PCa metastasis through its localization on lipid bilayers where it facilitates nucleocytoplasmic shuttling of EGFR signaling molecules and also regulates microRNA sorting in exosomes. These molecular functions of EWI‐2 result in downstream activation of the epidermal growth factor receptor‐mitogen‐activated protein kinase‐extracellular‐signal‐regulated kinase (EGFR‐MAPK‐ERK) signaling pathway and promote PCa cell migration.

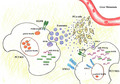

**Abbreviations**
Ab: antibody
Cav‐1: Caveolin‐1
cDNA: complementary DNA
EGFR: epidermal growth factor receptor
ERK: extracellular‐signal‐regulated kinase
ERM: ezrin/radixin/moesin
EV: extracellular vesicle
FITC: fluorescein isothiocyanate
FN: fibronectin
HE: , hematoxylin and eosin
IgSF: immunoglobulin superfamily
IHC: immunohistochemistry
LN: laminin
mAb: monoclonal antibody
MAPK: mitogen‐activated protein kinase
mRNA, messenger RNa
miR, microRNA
NE: nuclear envelope
NPC: nuclear pore complex
pAb: polyclonal antibody
PCa: prostate cancer
PIP: phosphatidylinositol phosphate
PRAD: prostate adenocarcinoma
PSA: prostate‐specific antigen
PTRF: Polymerase I and transcript release factor
PVDF: poly(vinylidene difluoride)
RT, room temperature
sgRNA: single guide RNA
siRNA, small interfering RNA
STORM: stochastic optical reconstruction microscopy

## 1 Introduction

The incidence rate of prostate cancer in almost all countries has increased in recent years [1]. However, the current tools used for the diagnosis of prostate cancer are not sensitive or specific for the detection [2], let alone for the precise distinction of metastatic prostate as well as the effective treatment of prostate cancer [3]. EWI‐2/PGRL, or EWI‐2 or CD316, is a binding partner of tetraspanins [4, 5]. It associates with transmembrane protein KAI1/CD82, which negatively regulates the metastasis potential of prostate cancer [6]. EWI‐2 is also physically associated with CD9, CD81 [5, 7] and other membrane proteins, such as integrins [8], immunoglobulin superfamily (IgSF) proteins [9], proteases and growth factor [10, 11]. As all of these membrane proteins regulate cancer cell movement, [12, 13, 14, 15, 16] their associations have functional impacts.

Recent studies have shown that CD9, CD81 and CD82 are enriched in extracellular vesicles (EV) and are often used as markers for exosomes [17]. Since EWI‐2 associates with these proteins, EWI‐2 may play an essential role in the composition of exosomes and correlate with its function in cancer diseases. Exosomes are nanosized lipid bilayer‐enclosed extracellular vesicles with diameters that range from 30 to 150 nm, and they are secreted constantly by most cells, including epithelial cells [18]. It has been demonstrated that exosomes are able to package and transfer proteins, DNA, messenger RNA (mRNA) and microRNA (miRNA) and to regulate cell–cell communication [18]. A previous study revealed that the *in situ* exosome miRNA detection may serve as a novel technique that provides methods of noninvasive liquid biopsies for diagnosing prostate cancer [19, 20, 21]. Therefore, the investigation of miRNA in exosomes may serve as a new tool for the diagnosis of prostate cancer which compensates for the shortcomings of current detection methods.

MicroRNA are small non‐coding RNA that can regulate post‐transcriptional gene expression [22]. MicroRNA can be loaded into extracellular vesicles, which allows those miRNA to circulate in the blood and avoid degradation by blood RNA enzyme [17]. The regulation of miRNA is not well studied. Several studies have shown that miRNA can be regulated by the control of the targets that they regulate, which form a feedback loop [23, 24, 25]. Given that exosomes are often formed from direct plasma membrane budding, accumulating evidence suggests that Cav‐1 is required for the regulation of packaging miRNA into exosomes [26, 27]. Membranaceous structure PTRF/cavin‐1 is newly reported to be involved as well in exosome secretion [27]. Other evidence has shown that pre‐miRNA are shuttled from the nucleus to the cytoplasm through nuclear pore complexes (NPC) located on the nuclear envelope (NE), which is important in human cancer [28]. As indicated above, because the lipid bilayer structure, including the plasma membrane and nuclear envelope, are crucial for the packaging and composition of exosomes, we proposed that the membrane protein EWI‐2, which associates with several exosome‐related protein markers, may function as a regulator of exosome formation and secretion.

A previous study has demonstrated that the cytoplasmic tail of EWI‐2 interacts specifically with phosphatidylinositol phosphates (PIP) on the cell membrane [29]. It is known that phosphoinositides are differentially localized in subcellular compartments such as the cell membrane and nuclear envelope [30], as well as the extracellular exosomes [31]. Interestingly, we identified that the location of EWI‐2 on the NE and exosomes in PC3 cells, which provides new insight into the functional investigation of EWI‐2 in prostate cancer.

Considering the hypothesis above, we investigated the function of EWI‐2 in the regulation of exosomes and signaling molecules, and further demonstrated the effect of EWI‐2 in the regulation of prostate cancer cell motility. Thus, we demonstrate here that EWI‐2 is highly expressed in exosomes and that the nuclear envelope, which governs the packaging of miRNA into exosomes, affects the cell membrane growth factor epidermal growth factor receptor‐mitogen‐activated protein kinase‐extracellular‐signal‐regulated kinase (EGFR‐MAPK‐ERK) signaling pathway and further leads to inhibited prostate cancer cell metastasis.

## 2 Materials and methods

### 2.1 Reagents

The antibodies used in this study are presented in Table 1, and special reagents are presented in Table 2.

**Table 1:** All antibodies used in this study

Clone name	Catalog number	Manufacturer
EWI‐2 polyclonal goat anti‐human IgG	AF3117	Novus (Novus Biologicals, Centennial, CO, USA)
Rabbit anti‐IGSF8 (Center) Polyclonal Antibody	abs112929	Absin Bioscience (Shanghai, China)
CD9 (D3H4P) Rabbit mAb	13403	Cell Signaling Technology (Danvers, MA, USA)
CD9 Rabbit Polyclonal Antibody	20597‐1‐AP	Proteintech (Wuhan Sanying, China)
CD81 mAb Antibody	66866‐1‐Ig	Proteintech (Wuhan Sanying, Wuhan, China)
Merlin/Ezrin/Radixin/Moesin (D1P8I) Rabbit mAb	23292	Cell Signaling Technology (Danvers, MA, USA)
EGFR mAb	610017	BD Biosciences (San Diego, CA, USA)
pTyr1068‐EGFR pAb	44‐788G	Thermo Fisher (Thermo Scientific, Rockford, IL, USA)
p44/42 MAPK mAb	612359	BD Biosciences (San Diego, CA, USA)
p‐p44/42 MAPK mAb	4370	Cell Signaling Technology (Danvers, MA, USA)
EGFR mAb [EP38Y] (Alexa Fluor 647)	ab192982	Abcam (Cambridge, MA, USA)
CD54/ICAM‐1 (E3Q9N) Rabbit mAb	67836	Cell Signaling Technology (Danvers, MA, USA)
Flotillin‐1 (D2V7J) Rabbit mAb	18634	Cell Signaling Technology (Danvers, MA, USA)
EpCAM (E6V8Y) Rabbit mAb	93790	Cell Signaling Technology (Danvers, MA, USA)
Annexin A1 (D5V2T) Rabbit mAb	32934	Cell Signaling Technology (Danvers, MA, USA)
EEA1 (C45B10) Rabbit mAb	3288	Cell Signaling Technology (Danvers, MA, USA)
Lamin A/C (4C11) Mouse mAb (Alexa Fluor 488 conjugate)	8617	Cell Signaling Technology (Danvers, MA, USA)
Lamin A/C (4C11) Mouse mAb	4777	Cell Signaling Technology (Danvers, MA, USA)
Rabbit Anti‐Caveolin‐1 antibody	ab2910	Abcam (Cambridge, MA, USA)
Rabbit Anti‐PTRF antibody	ab88213	Abcam (Cambridge, MA, USA)
Anti‐Integrin alpha V antibody [272‐17E6]	ab16821	Abcam (Cambridge, MA, USA)
Merlin (D3S3W)	12888	Cell Signaling Technology (Danvers, MA, USA)
HER2/ErbB2 (29D8) Rabbit mAb	2165	Cell Signaling Technology (Danvers, MA, USA)
Akt Antibody	9272	Cell Signaling Technology (Danvers, MA, USA)
Phospho‐Akt (Ser473) (D9E)	4060	Cell Signaling Technology (Danvers, MA, USA)
N‐Cadherin (D4R1H)	13116	Cell Signaling Technology (Danvers, MA, USA)
E‐Cadherin (4A2) Mouse mAb	14472	Cell Signaling Technology (Danvers, MA, USA)
Vimentin (D21H3) Rabbit mAb	5741	Cell Signaling Technology (Danvers, MA, USA)
β‐Catenin (D10A8) Rabbit mAb	8480	Cell Signaling Technology (Danvers, MA, USA)
Cleaved Caspase‐3 (Asp175)	9661	Cell Signaling Technology (Danvers, MA, USA)
GAPDH mAb	G9545‐100UL	Sigma‐Aldrich (Merck, KGaA, Darmstadt, Germany)
β‐actin mAb	A5441‐100UL	Sigma‐Aldrich (Merck, KGaA, Darmstadt, Germany)
Alexa Fluor 488‐conjugated phalloidin	A12379	Thermo Fisher (Thermo Scientific, Rockford, IL, USA)
Goat anti‐Mouse IgG (H+L) Highly Cross‐Adsorbed Secondary Antibody, Alexa Fluor Plus 488	A32723	Thermo Fisher (Thermo Scientific, Rockford, IL, USA)
Goat anti‐Rabbit IgG (H+L) Highly Cross‐Adsorbed Secondary Antibody, Alexa Fluor Plus 488	A32731	Thermo Fisher (Thermo Scientific, Rockford, IL, USA)
Goat anti‐Mouse IgG (H+L) Highly Cross‐Adsorbed Secondary Antibody, Alexa Fluor Plus 647	A32728	Thermo Fisher (Thermo Scientific, Rockford, IL, USA)
Goat anti‐Rabbit IgG (H+L) Highly Cross‐Adsorbed Secondary Antibody, Alexa Fluor Plus 647	A32733	Thermo Fisher (Thermo Scientific, Rockford, IL, USA)
Donkey anti‐Goat IgG (H+L) Highly Cross‐Adsorbed Secondary Antibody, Alexa Fluor Plus 647	A32849	Thermo Fisher (Thermo Scientific, Rockford, IL, USA)
Donkey anti‐Goat IgG (H+L) Highly Cross‐Adsorbed Secondary Antibody, Alexa Fluor Plus 488	A32814	Thermo Fisher (Thermo Scientific, Rockford, IL, USA)
Anti‐Mouse IgG (Fab specific)–Peroxidase antibody	A9917	Sigma‐Aldrich (Merck, KGaA, Darmstadt, Germany)
Anti‐Rabbit IgG (whole molecule)–Peroxidase antibody	A0545	Sigma‐Aldrich (Merck, KGaA, Darmstadt, Germany)
Anti‐Mouse IgG (whole molecule)–FITC antibody	F2012	Sigma‐Aldrich (Merck, KGaA, Darmstadt, Germany)
Anti‐Rabbit IgG (whole molecule)–FITC antibody	F0382	Sigma‐Aldrich (Merck, KGaA, Darmstadt, Germany)
Peroxidase‐Conjugated Rabbit anti‐Goat IgG (H+L)	ZB‐2306	ZSGB‐BIO (Beijing, China)

**Table 2:** All special reagents used in this study

Reagents	Catalog number	Manufacturer
Gefitinib	SML1657	Sigma‐Aldrich (Merck, KGaA, Darmstadt, Germany)
Minute™ Nuclear Envelope Protein Extraction kit	NE‐013	Invent (Plymouth, MN, USA)
Lipofectamine™ RNAiMAX Transfection Reagent	13778075	Thermo Fisher (Thermo Scientific, Rockford, IL, USA)
Lipofectamine™ 3000 Transfection Reagent	L3000008	Thermo Fisher (Thermo Scientific, Rockford, IL, USA)
Opti‐MEM serum‐reduced medium	11068‐021	Thermo Fisher (Thermo Scientific, Rockford, IL, USA)
Fibronectin Human Protein, Native	PHE0023	Thermo Fisher (Thermo Scientific, Rockford, IL, USA)
Human Recombinant Laminin 111	LN111‐02	BioLamina (Sundbyberg, Sweden)
Corning® Matrigel® Basement Membrane Matrix	354234	Corning (New York, NY, USA)
miRNeasy Mini kit	217004	Qiagen (Germantown, MD, USA)
NEXTFLEX® Small RNA‐Seq Kit v3	NOVA‐5132‐05	Bioo Scientific (Austin, TX, USA)
AMPure XP beads	A63881	Beckman (Indianapolis, IN, USA)
Qubit HS DNA kit	Q32854	Thermo Fisher (Thermo Scientific, Rockford, IL, USA)
2100 DNA high‐sensitivity kit	5067‐4626	Agilent (Santa Clara, CA, USA)
HiSeq SBS Kit v4	FC‐401‐4003	Illumina (San Diego, CA, USA)
HiSeq Cluster Kit v4	GD‐401‐4001	Illumina (San Diego, CA, USA)
miRNA First Strand cDNA Synthesis kit (Tailing Reaction)	B532451	Sangon Biotech (Shanghai, China)
MicroRNA qPCR Kit (SYBR Green Method)	B532461	Sangon Biotech (Shanghai, China)
miDETECT A Track cel‐miR‐39‐3p Forward Primer	miRA0000010‐1‐100	RiboBio (Guangzhou, China)
cel‐miR‐39‐3p Standard RNA, HPLC, 1nmol	miRB0000010‐3‐1	RiboBio (Guangzhou, China)
hEGF	E9644	Sigma‐Aldrich (Merck, KGaA, Darmstadt, Germany)
pHrodo™ Green Epidermal Growth Factor (EGF) Conjugate	P35375	Thermo Fisher (Thermo Scientific, Rockford, IL, USA)
Live Cell Imaging Solution	A14291DJ	Thermo Fisher (Thermo Scientific, Rockford, IL, USA)

### 2.2 Cell culture, EWI‐2 knockout and overexpression

PC3 and Du145 human metastatic prostate cancer cells were obtained from ATCC and cultured in complete Dulbecco’s modified Eagle’s medium (DMEM) containing 10% (FBS), glutamine and penicillin/streptomycin.

EWI‐2‐null PC3 and Du145 cells were generated using CRISPR/Cas9 technology. For PC3 and Du145 cells, four single guide RNA (sgRNA) targeting multiple EWI‐2 locus (Table 3) were cloned into the PX458 vector to generate a PX458‐EWI‐2 knockout construct. Cells were seeded at a density of 0.5–1 × 10^5^ cells/well in six‐well plates and transfected with PX458‐EWI‐2‐knockout construct (2.5 µg/well) using Lipofectamine 3000. The cells expressing GFP but not EWI‐2 were collected by flow cytometry at 48–72 h after transfection and cultured in complete medium. The second round of sorting was performed for GFP‐negative and EWI‐2‐negative cells. Sorting was repeated until 100% purity of EWI‐2 negative cells was reached. Cells were then examined for EWI‐2 expression with western blotting.

**Table 3:** The sgRNAs used in this study for EWI‐2 knockout in PC3 cells

sgRNAs	EWI2 sgRNA1‐Forward:	EWI2 sgRNA1‐Reverse:
	CACCGGACGCGGTACAAGGGCCCCTC	AAACGAGGGGCCCTTGTACCGCGTC
	**EWI2 sgRNA2‐Forward:**	**EWI2 sgRNA2‐Reverse:**
	CACCGGACTTCGAGTGGTTCCTGTAT	AAACATACAGGAACCACTCGAAGTC
	**EWI2 sgRNA3‐Forward:**	**EWI2 sgRNA3‐Reverse:**
	CACCGGTGTCTTCAAGTCCCGAGTGG	AAACCCACTCGGGACTTGAAGACAC
	**EWI2 sgRNA4‐Forward:**	**EWI2 sgRNA4‐Reverse:**
	CACCGGACTCATAAATGCCGGCATCC	AAACGGATGCCGGCATTTATGAGTC

EWI‐2 mock and wild‐type were constructed into a eukaryotic expression vector pDest‐C‐GFP. Briefly, the full‐length complementary DNA (cDNA) encoding human wild‐type EWI‐2 were inserted into the pDest‐C‐GFP vector through the *Asis*I and *Mlu*I sites. The EWI‐2 coding information was then confirmed by DNA sequencing. PC3 cells were transfected with GFP fusion constructs using Lipofectamine 3000 and selected with puromycin at a concentration of 5 µg·mL^–1^. The EWI‐2‐positive clones were collected by cell sorting, and the stable transfectants of EWI‐2 wild‐type and mock were used in the following experiments.

### 2.3 EWI‐2 silencing

A small interfering RNA (siRNA) oligo against the target sequence GUUCUCCUAUGCUGUCUU [9] of EWI‐2 mRNA was used to silence EWI‐2 expression. PC3 cells (1 × 10^4^ cells per well of a 24‐well plate) were transfected with the siRNA using lipofectamine RNAiMAX (Thermo Scientific, Rockford, IL, USA). The experiments were performed 2 days posttransfection. PC3 cells transfected with EWI‐2 siRNA were named as PC3 KD. The cells transfected with nonspecific control siRNA were named PC3 NEG.

### 2.4 Flow cytometry analysis

After stable transfectants were established, cells were incubated with primary antibodies, followed by washing and subsequent staining with FITC‐conjugated secondary antibodies (Table 1). After staining, cells were examined with an automated Four‐Color Benchtop flow cytometer (BD Biosciences, San Diego, CA, USA) with CellQuest program (BD Biosciences). The flow cytometry data were analyzed with the flowjo 7.6.1 program.

### 2.5 Exosome isolation

Cells at 50–60% confluence were detached with 2 mm EDTA/PBS, washed 2 times with PBS and then cultured in DMEM supplemented with 5% Exo‐FBS (SBI, Palo Alto, CA, USA) until reaching 80–90% confluence. The cell culture supernatants were collected and centrifuged at 300 ***g*** for 10 min, 2000 ***g*** for 10 min, 10 000 ***g*** for 30 min and 100 000 ***g*** for 70 min. Exosomes were washed with PBS at 100 000 ***g*** and centrifuged for 70 min, and the pellet containing exosomes preserved.

### 2.6 RNA extraction, real‐time PCR analysis and miRNA mimic transfection

Total miRNA of exosomes was extracted by miRNeasy Mini kit (Qiagen, MD, USA). A miRNA First Strand cDNA Synthesis kit (Tailing Reaction) (Sangon Biotech, Shanghai, China) was used to synthesize miRNA cDNA from the total miRNA of exosomes. The synthesized exogenous reference cel‐miR‐39‐3p standard RNA (1 pmol per sample; RiboBio, Guangzhou, China) was added to the total RNA of exosomes in advance. Real‐time PCR for miRNA was carried out using a MicroRNA qPCR Kit (SYBR Green Method) (Sangon Biotech). The miRNA levels in exosomes were normalized against the exogenous reference cel‐miR‐39. Sequences of primers (Sangon Biotech) used for real‐time PCR in this study are listed in Table 4. The miR‐29a、miR‐149‐3p and miR‐3934‐5p as well as miRctrl mimics (2 μM per sample) were synthesized and transfected with lipofectamine RNAiMAX (Thermo Scientific, Rockford, IL, USA). The sequences were presented in Table 5.

**Table 4:** RT‐PCR primer used for detection of miRNA expression in the exosomes of PC3 cells

miRNA	RT‐PCR Primer
miR‐149‐3p	TATATAGGGAGGGACGGGGGC
miR‐29a‐5p	CCGCGACTGATTTCTTTTGGTGTTCAG
miR‐3934‐5p	TCAGGTGTGGAAACTGAGGCAG
cel‐miR‐39‐3p	miDETECT A Track cel‐miR‐39‐3p Forward Primer (RiboBio, China)

**Table 5:** The miRNA mimics used in this study

miRNA	Mimics
miR‐149‐3p	AGGGAGGGACGGGGGCUGUGC
miR‐29a‐5p	ACUGAUUUCUUUUGGUGUUCAG
miR‐3934‐5p	UCAGGUGUGGAAACUGAGGCAG

### 2.7 Nuclear envelope protein extraction

Cells were detached and 1.0–2.0 × 10^7^ cultured cells were collected with low‐speed centrifugation. NE was then extracted following instructions from the manufacturer of the kit (Invent, Plymouth, MN, USA). The cell pallet was washed with cold PBS, resuspended in 0.5 mL buffer A, incubated on ice for 10 min and vortexed for 10–20 s. Cell suspension was transferred to a filter cartridge and centrifuged at 14 000 ***g*** for 30 s. The supernatant was then removed, and the pellet was washed in 1 mL cold PBS, vortexed for 10 s and centrifuged at 500 ***g*** for 2 min. PBS was removed and 300 µL buffer B was added to the pellet, vortexed for 10 s, incubated on ice for 5 min, and then vortexed for another 10 s. Five minutes of incubation/10 s of vertex was repeated and then centrifuged at 5000 ***g*** and 4 °C for 5 min. The supernatant was transferred to new tube, 0.8 mL cold PBS was added and the tube was inverted approximately 10 times. The solution was then centrifuged at 16 000 ***g*** and 4 °C for 15 min. The pallet was isolated nuclear envelope and then dissolved with denaturing protein solubilization reagent (Invent) for downstream experiments.

### 2.8 Human tissue microarray assay

Premade formalin‐fixed, paraffin‐embedded (FFPE) tumor microarrays (TMA) for human prostate tumor tissues were from Outdo (Outdo Biotech, Shanghai, China). The experiments were undertaken with the understanding and written consent of each subject and the study methodologies conformed to the standards set by the Declaration of Helsinki. The study methodologies were approved by the West China Hospital of Sichuan University Biomedical Research Ethics Committee. Both the tumor tissues and noncancerous tissues were histologically confirmed. Tissues were stained with hematoxylin & eosin (HE). Images were acquired with NanoZoomer‐SQ and analyzed by NanoZoomer digital pathology software.

### 2.9 Western blot analysis

Cells were lysed with RIPA buffer on ice for 30 min. After centrifugation at 13 000 ***g*** for 15 min, soluble portions of the cell lysates were separated by SDS/PAGE and then electrically transferred to nitrocellulose/poly(vinylidene difluoride) (PVDF) membranes. The membranes were blocked with fat‐free milk and then blotted sequentially with primary Abs (Table 1) and horseradish peroxidase‐conjugated secondary antibody, followed by chemiluminescence.

### 2.10 Cell movement assays

Directional solitary cell migration was examined with a Transwell migration assay. Transwell inserts were coated with either fibronectin or laminin 111 and then blocked with heat‐inactivated BSA. Six thousand cells were suspended in DMEM/1% BSA, loaded into inserts, and incubated at 37 °C and 5% CO_2_ for 3–6 h.

Cell invasion was examined with Transwell inserts coated with Matrigel. The cells were added on the gel and incubated overnight at 37 °C and 5% CO_2_. The cells that invaded through the inserts were stained with 0.1% crystal violet solution and then imaged. Cell number was counted using imagej software.

### 2.11 Confocal fluorescence microscopy

Cells were cultured in complete DMEM for 48–72 h, fixed with 3% paraformaldehyde, and permeabilized with 0.1% Brij98. Then, cells were incubated sequentially with primary antibodies and fluorochrome‐conjugated secondary antibody. Cells were then examined and imaged with a Leica SP2 MP confocal microscope equipped with a 63x Plan APO 1.4 NA oil immersion objective. imagej was used for image analysis.

### 2.12 Super‐resolution imaging

Stochastic optical reconstruction microscopy (STORM) was used for examining EGFR distribution. Cells were cultured in the 8‐well chambered coverglass (Thermo Scientific) for 12–24 h, treated with human EGF(25 nm)/PBS for 3 min, and washed once with PBS. The cells were then fixed with 3% glyoxal solution at rom temperature (RT) for 1 h and washed sequentially with 0.1% NaBH_4_/PBS for 5 min and 10 mm Tris/PBS for 2 × 5 min at RT, followed by blocking with 5% BSA/0.05% Triton X‐100/PBS for 15 min. The cells were labeled with 4 μg·mL^–1^ of Alexa Fluor 647‐conjugated EGFR antibody in 2% BSA/0.05% Triton X‐100/PBS at RT for 1 h, washed with 2% BSA/0.05% Triton X‐100/PBS at RT 3x for 5 min each time and with PBS for 5 min, and imaged with an N‐STORM imaging modality (Nikon). Images were analyzed sequentially with NIS‐Elements AR and graphpad prism 5.0.

### 2.13 Experimental metastasis assay

PC3 cells (2.0 × 10^6^ cells/administration) were injected into the tail veins of NOG mice (NOD. Cg‐*Prkdc^SCID^ Il2rg^tm1Sug^*/JicCrl), which were originally obtained from the Japan Central Institute of Experimental Animals. The mice were maintained in the Experimental Animal Center of the Science and Technology Base of West China Hospital, Sichuan University. All animals were kept in cages in the animal room, ambient temperature was 23 ± 2 °C; 5 mice per cage, with food and water *ad libitum*. Light and dark time alternated for 12 h. All institutional and national guidelines for the care and use of laboratory animals were followed. The experiments were performed in accordance with the experimental animal ethics committee of West China Hospital as well. The licence number approved for the experiments was 2019267A.

The mice were weighed every 3 days after the injection and euthanized when the loss of bodyweight reached 20% and the mice became less physically responsive. The lungs and livers were collected, macrometastatic lesions were counted with the naked eye and measured, and the organs were sectioned and stained with HE.

### 2.14 Subcutaneous tumor formation assay

PC3 cells (4.0 × 10^6^ cells/administration) were injected subcutaneously in NOD SCID mice (NOD.CB17‐Prkdc^scid^/NcrCrl), which were obtained originally from Charles River Laboratories. The housing and handling conditions of the mice were consistent with the method above. The experiments were performed in accordance with the experimental animal ethics committee of West China Hospital, Sichuan University. The licence number approved for the experiments was 2020038A.

The mice were weighed every 3 days after the injection and euthanized when tumor size reached 1.0–1.5 cm. The tumors were collected and weighed, and the size measured and then quantified with graphpad prism software.

### 2.15 MiRNA microarray analysis

Total RNA was extracted using a miRNeasy Mini kit (Qiagen, MD, USA). The pellets obtained from the exosome isolation were lysed by adding 700 µL of lysis solution and mixed using vortex, then 140 µL chloroform was added to the vial and gently mixed. After staying in room temperature for 2–3 min, the mixture was centrifuged at 12 000 ***g*** and 4 °C for 15 min. Ethanol 450–600 µL was then added to the mixture in the vial, which was then transferred to the mini spin column and centrifuged for 15 s at ≥ 8000 ***g***. Next, 700 µL Buffer RWT and 500 µL Buffer RPE were added in turn and centrifuged for 15 s at ≥ 8000 ***g***; 500 µL Buffer RPE was added and the column was centrifuged at ≥ 10 000 ***g*** for 2 min. The column was then transferred to a new EP tube and dried at RT for 3–5 min, followed by the addition of 24–40 µL DEPC H_2_O and centrifugation for 1 min at ≥ 8000 ***g***. The RNA was collected in an EP tube.

The miRNA Library was constructed with the NEXTFLEX® Small RNA‐Seq Kit v3 (Bioo Scientific Corp., Austin, TX USA), AMPure XP beads (Beckman, Indianapolis, IN, USA), Qubit HS DNA kit (Thermo Fisher) and 2100 DNA high‐sensitivity kit (Agilent, Santa Clara, CA, USA) following standard protocols. An Agilent 2100 Bioanalyzer high‐sensitivity DNA assay was used to detect the fragment distribution range of the library; the main peak of the library is about 150 bp. MicroRNA sequencing was then performed with a HiSeq SBS Kit v4 (Illumina, San Diego, CA, USA), HiSeq Cluster Kit v4 (Illumina.) on an Illumina HiSeq 2500 and sequenced with single‐end 50 bp.

### 2.16 The miRNA target prediction

The miRNA target prediction and analysis were performed with the algorithms from

TargetScan (http://www.targetscan.org/), miRanda (http://www.miranda.org/), PITA (https://genie.weizmann.ac.il/pubs/mir07/mir07_data.html), KEGG (https://www.genome.jp/kegg/pathway.html).

### 2.17 TCGA database

The expression of EWI‐2/Igsf8 in prostate adenocarcinoma was searched in the TCGA database: (http://ualcan.path.uab.edu/index.html). The data were presented on nodal metastasis status and Gleason score.

### 2.18 Endocytosis assays

For Ab‐based endocytosis of EGFR, PC3 NEG and KO cells were plated on coverslips at 50% confluence. Cells were incubated with EGFR mAb (1 μg·mL^–1^) at 4 °C for 1 h. After the incubation, the cells were washed three times with ice‐chilled PBS. Afterwards, 37 °C pre‐warmed DMEM was added. Cells were incubated at 37 °C for 15–20 min. After incubation, the antibodies that were bound to the cell surface but not internalized were removed by three washes of 0.1 m glycine/1.0 m NaCl solution (pH 2.5). The cells were then fixed with 3% paraformaldehyde, permeabilized with 0.1% Brij98 and incubated with secondary antibody for immunofluorescence.

For EGF‐based endocytosis of EGFR, PC3 NEG and KO cells were plated and kept on ice for 10 min and then washed with cold live cell imaging solution (LCIS, HEPES‐based buffer at pH 7.4, supplemented with 20 mm glucose and 1% BSA). We added 2 μg·mL^–1^ pHrodo green EGF conjugate (Thermo Fisher) in LCIS (Live Cell Imaging Solution) containing 20 mm glucose and 1% BSA, to the cells and incubated them for 15–20 min at 37 °C. Cells were washed with LCIS and fixed with 3% paraformaldehyde. Cells were then imaged under an N‐STORM imaging modality (Nikon) confocal microscope with an excitation of 509 nm and emission 518 nm, and a 63x Plan APO 1.4 NA oil immersion objective. The images were analyzed using imagej software.

## 3 Results

### 3.1 EWI‐2 is highly expressed in extracellular vesicles in prostate cancer cells

**Fig. 1:**
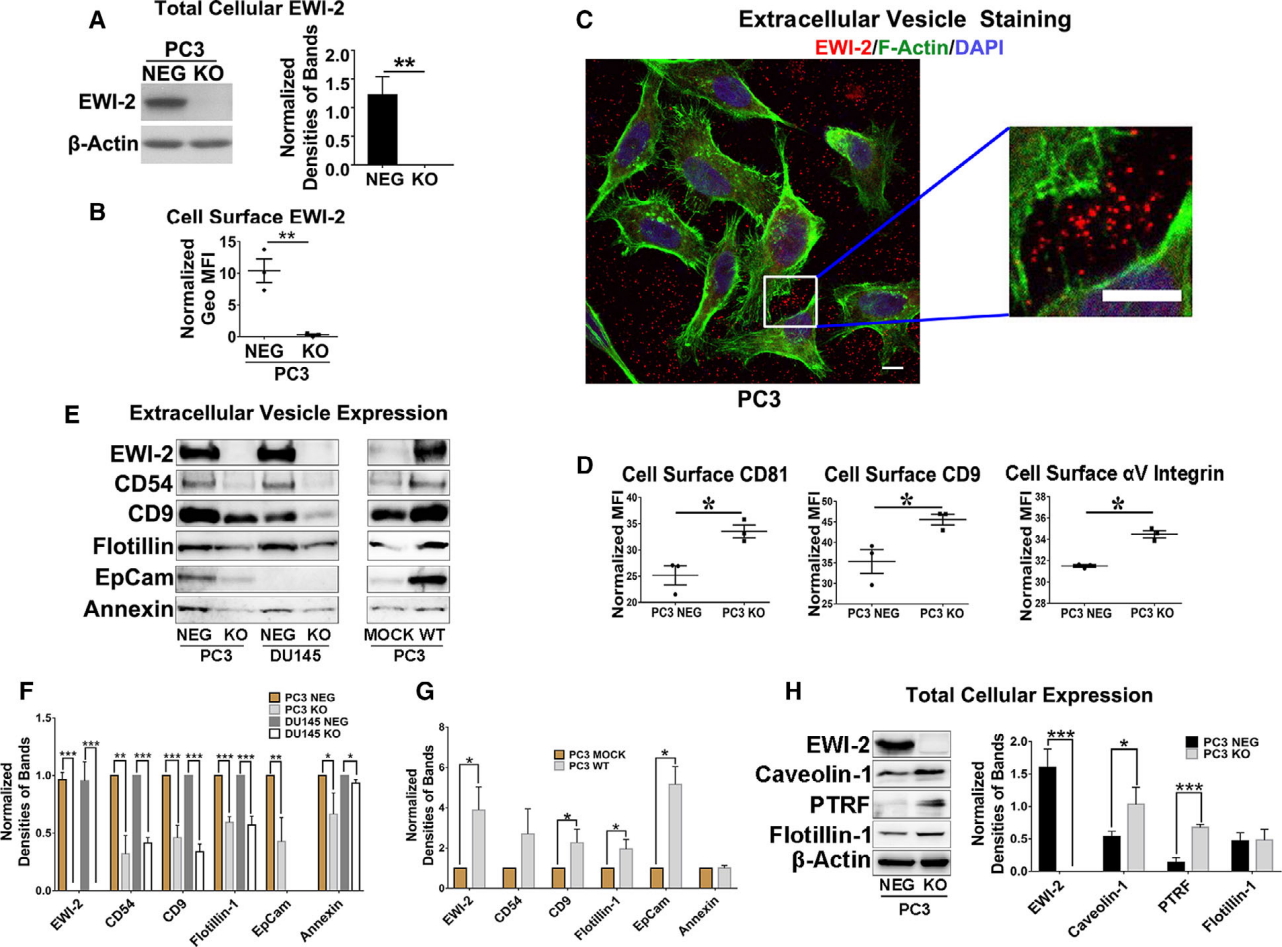
EWI‐2 expression in extracellular vesicles. (A) Western blot analysis of EWI‐2 protein levels in PC3 cells upon EWI‐2 knockout. Actin serves as a protein loading control. The normalized band densities were quantified with Student’s *t*‐test and are presented as mean ± SD (*n* = 3 individual experiments). **P < 0.01. (B) EWI‐2‐CRISPR/Cas9 knockout efficiency in PC3 cells, as analyzed with flow cytometry. Student’s *t*‐test was used for statistical analysis on fluorescence intensity (MFI) (mean ± SD, *n* = 3 individual measurements). **P < 0.01. (C) Extracellular staining of EWI‐2 in PC3 cells. After the cells were fixed, permeabilized and stained for EWI‐2 mAb, F‐actin with phalloidin, and nucleus with DAPI, immunofluorescence images were captured with confocal fluorescence microscopy. Scale bar: 10 µm. (D) Cell surface CD9, CD81 and integrin αV expression in PC3 cells, as analyzed with flow cytometry. Student’s *t‐*test was used for statistical analysis on MFI (mean ± SD, *n* = 3 individual measurements; *P < 0.05). (E) The exosomes were extracted and examined with western blot analysis. The membrane were probed with EWI‐2 and exosome marker antibody (anti‐CD54, anti‐CD9, anti‐flotillin, anti‐EpCam and anti‐annexin antibody). (F) The normalized band densities of PC3‐EWI‐2‐CRISPR/Cas9 knockout cells and DU145‐EWI‐2‐CRISPR/Cas9 knockout cells in (E) were quantified with Student’s *t*‐test and presented as mean ± SD (*n* = 3 individual experiments). *P < 0.05; **P < 0.01; ***P < 0.001. (G) The normalized band densities of PC3‐EWI‐2‐overexpression cells were quantified with Student’s *t*‐test and presented as mean ± SD (*n* = 3 individual experiments). *P < 0.05. (H) Western blot analysis of cellular expression of Caveolin‐1, PTRF (Cavin‐1) and Flotillin‐1, together with actin as a loading control of whole‐cell lysates. The band density of KO cells in each individual experiment was normalized with the density of NEG cells. The normalized band densities were quantified with Student’s *t*‐test and presented as mean ± SD (*n* = 3 individual experiments). *P < 0.05; ***P < 0.001.

EWI‐2 was previously demonstrated to be expressed on the plasma membrane as a transmembrane protein [5, 9]. Because it had a direct association with several exosome markers, such as CD9, CD81 and CD82, we carried out exosome extraction and analysis for the prostate cancer cell lines PC3 and Du145 cells with EWI‐2 CRISPR/Cas9 knockout (Fig. 1A,B,E) as well as the overexpression of EWI‐2 in PC3 cells (Fig. 1E). From immunofluorescence staining, we detected EWI‐2 staining outside of the PC3 cells with diffuse distribution, which indicated that there was a large amount of small particles released from the cells containing EWI‐2(Fig. 1C). Exosomes can be secreted by almost all kinds of cell types and play important roles in communication between cells. Because we found extracellular staining of EWI‐2, we investigated whether EWI‐2 indeed existed in exosomes and contributed to the effect of exosomes. Based on this observation and assumption, we further extracted the exosomes from the cell culture medium of prostate cancer cells, performed western blot analysis and confirmed their identity using positive exosome markers (Annexin, EpCam, CD54, CD9 and flotillin). Interestingly, there was a relative high amount of EWI‐2 enrichment in exosome components, and the EWI‐2 expression level in exosomes was positively correlated with the levels of other exosome markers, such as flotillin, CD9 and EpCam, in both EWI‐2 knockout and EWI‐2‐overexpressing cells (Fig. 1F,G). Although the expression levels of exosome markers were all decreased based on EWI‐2 knockout, however, together with the nanoparticle tracking analysis in Fig. S1 (Appendix S1), from the same amount of cell culture medium extraction of exosomes, no obvious downregulation of size or concentration was observed, which indicated that the function of EWI‐2 in the regulation of exosome might not be a simple effect on the change of vesicle numbers but the content screening into the exosomes. These results indicate that EWI‐2 indeed exists in the exosomes and may play a similar role to the exosome markers; this needs further investigation. Therefore, in the following experiments, we mainly study whether EWI‐2 regulates the invasiveness of prostate cancer cells by influencing the biological function of exosomes, and we specifically elaborate the important role of exosomes in the invasiveness of prostate cancer cells.

### 3.2 EWI‐2 regulates the sorting of multiple miRNA into exosomes

In accordance with the previous observations that EWI‐2 is enriched in exosomes and the foregoing results that the role of EWI‐2 could regulate prostate cancer cell metastasis [6], we assumed that EWI‐2 could shuttle the miRNA enriched in exosomes in PC3 cells. As Cav‐1 is important for the sorting of miRNA into exosomes, we tested the Cav‐1 and PTRF expression in PC3 NEG and EWI‐2 KO cells. EWI‐2 depletion upregulated Cav‐1 expression. Interestingly, although PTRF was not supposed to be expressed in the PC3 cell line, PTRF expression significantly increased in PC3 EWI‐2 KO cells(Fig. 1H). However, the molecular scaffold protein flotillin‐1, which is important for the export of exosomes from cells, was not significantly changed (Fig. 1H). These results imply that the capacity of miRNA sorting to the exosomes may be elevated in EWI‐2 KO cells.

**Fig. 2:**
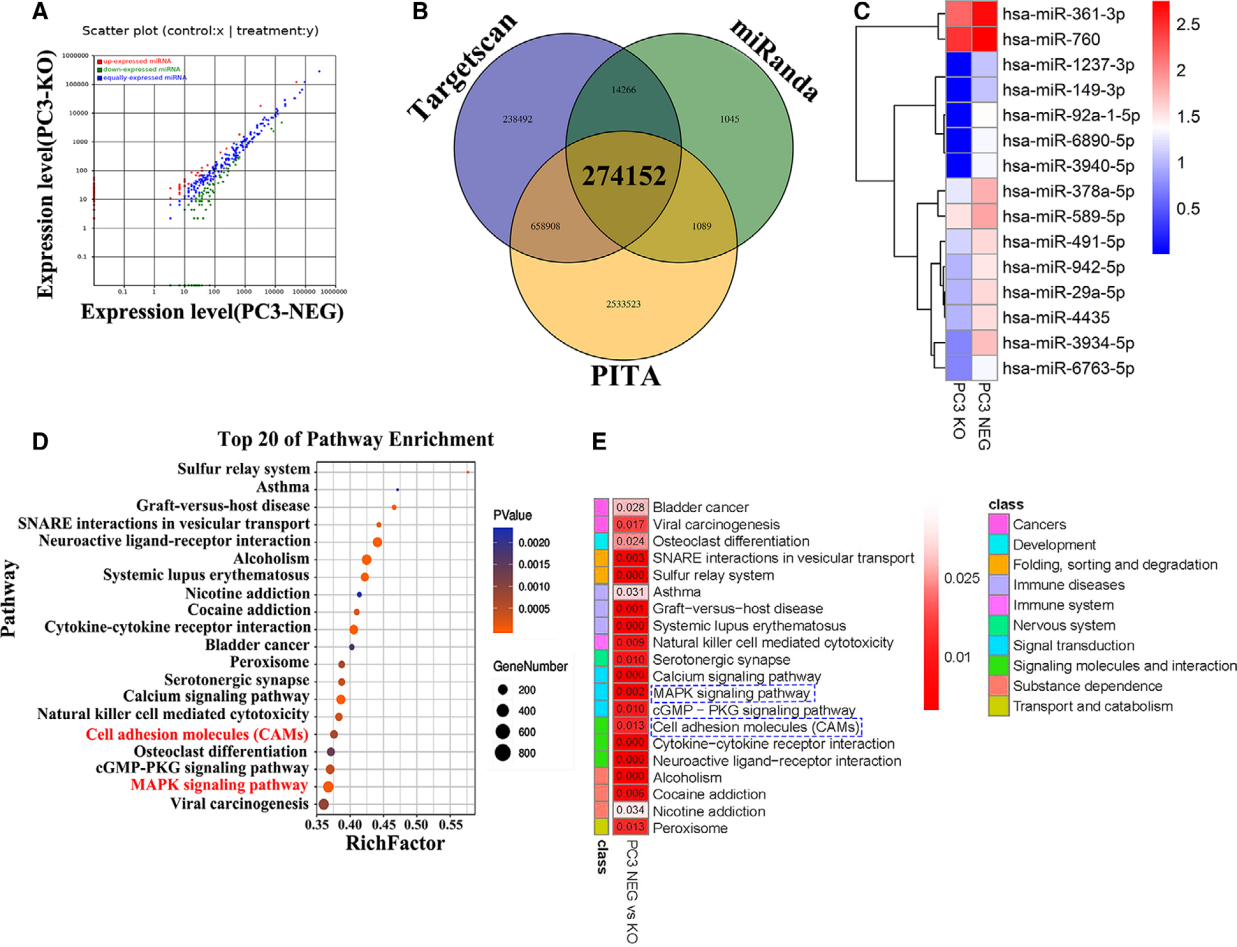
EWI‐2 regulates miRNA expression profile in the exosomes from prostate cancer cells. (A) Scatter plot of the differential gene expressions in the exosomes of prostate cancer cells with significant difference. (B) Venn diagram of exosomal miRNA shared among three datasets. Three different color ellipses in the figure represent the number of differential exosomal miRNA in three different datasets, and the middle part represents the cross‐section of the three dataset analysis results. (C) Heat map showed the significantly changed expressed miRNA based on EWI‐2 knock out with EGFR as potential target. In the heat map, red color shows upregulated miRNA with high‐fold change and blue color shows the downregulated miRNA with high‐fold change. (D,E) The top 20 pathway enrichments in prostate cancer cells. The pathway enrichment differentiating between PC3 NEG and PC3 KO cell lines based on the exosomal miRNA profile analysis. Shown as variable importance in the projection (Rich factor score and *P*‐value). Relative pathway abundance is indicated in the bar (E), with red representing the correlation of relative pathway.

The exosomes of PC3 cells were then extracted and analyzed. Nanoparticle tracking analysis was utilized to evaluate the size distribution of exosomes; the mean size of extracellular vesicles from PC3 NEG cells was found to be 141.5 and 164.9 nm in the PC3 KO group, with most extracellular vesicles distributed within the range of the exosome diameter (30–150 nm). The most abundant extracellular vesicles were approximately 146 nm in the PC3 NEG group and 151 nm in the PC3 KO group in diameter (Fig. S1). The exosomes extracted from the PC3 NEG and KO cells were then used for miRNA expression profiling. On EWI‐2 knockout, 155 significantly expressed miRNA were revealed, with more than a twofold expression change: 100 downregulated and 55 upregulated miRNA, as shown in the scatter plot (Fig. 2A). These aberrantly expressed miRNA may participate in prostate cancer metastasis. To further screen out prostate cancer‐associated target genes, Venn analysis was performed to obtain the cross‐section of the differential genes in the three datasets (miRanda, TargetScan, PITA). In all, 274152 target genes were shared by all five datasets (Fig. 2B). Some aberrantly expressed miRNA with EGFR as predicted target were found in the miRNA microarray assay. From the downregulated miRNA, we found 15 miRNA with EGFR as the predicted target gene, as indicated in the heat map (Fig. 2C).

To analyze further the pathways related to the differential expression of miRNA caused by EWI‐2 knockout, KEGG analysis was performed based on the results of miRNA microarray assay. The top 20 pathway enrichments were selected by Rich factor with mitogen‐activated protein kinase (MAPK) signaling and cell adhesion molecule signaling at high Rich factor values (Fig. 2D,E). Interestingly, the cell–cell adhesion analysis also confirmed the results, which showed that in PC3 KO cells, the cell–cell adhesion capacity was obviously weakened (Supporting Information Fig. S2D,E, Appendix S1). As reported elsewhere, EGFR and the extracellular matrix play crucial roles in cell adhesion [32]. These results imply that EGFR may serve as valued candidate for the exosomal miRNA target gene, which plays an important role in the regulation of prostate cancer metastasis.

**Fig. 3:**
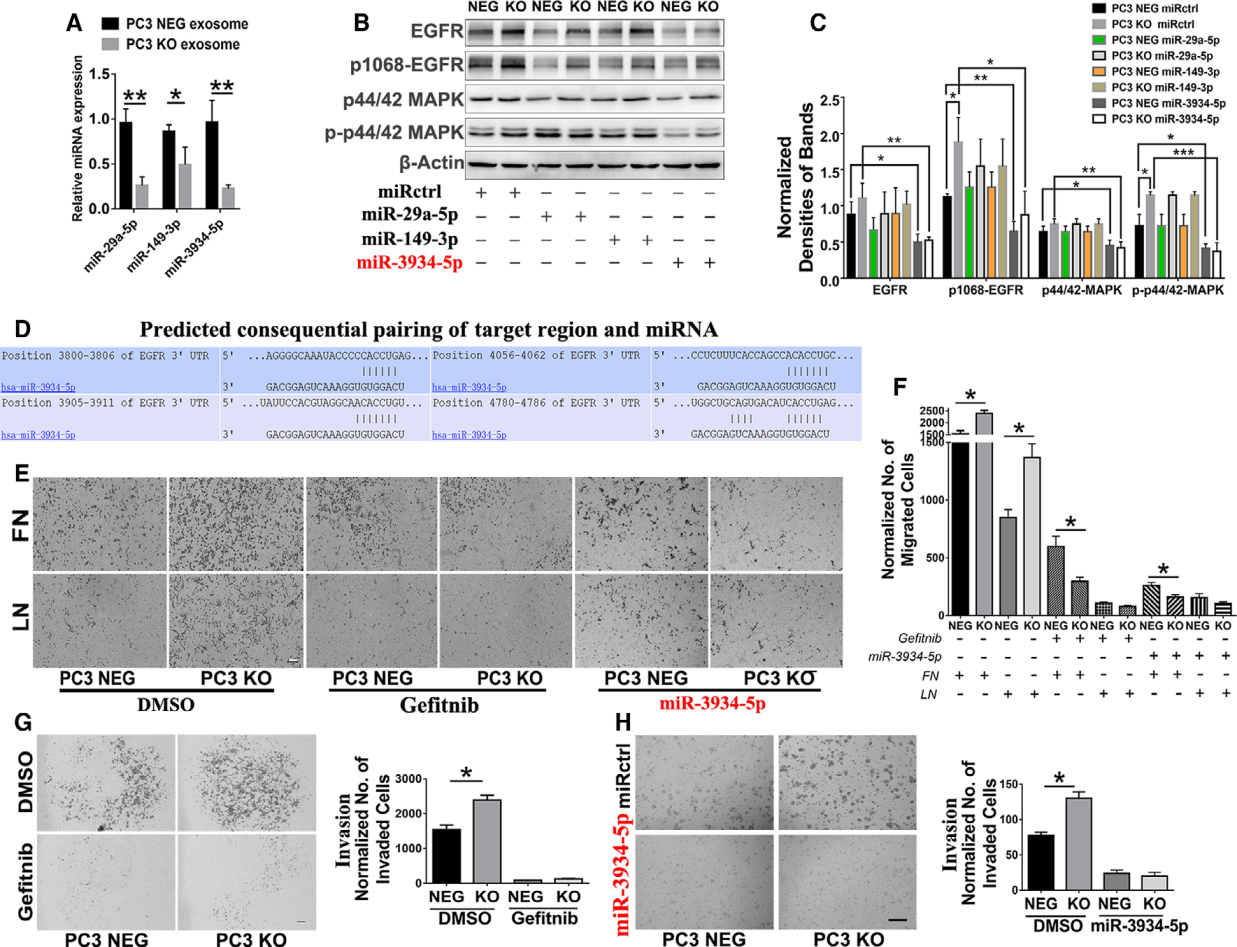
EWI‐2 removal promotes tumor cell motility by regulating the enrichment of exosomal miR‐3934‐5p. (A) Exosomes from PC3 NEG and KO cells were purified and miRNA of exosomes was extracted. Real‐time PCR for miRNA was carried out and the miR‐29a, miR‐149‐3p and miR‐3934‐5p expression levels in exosomes were detected. The relative miRNA expression level was quantified with Student’s *t*‐test and presented as mean ± SD (*n* = 3 individual experiments). *P < 0.05; **P < 0.01. (B) PC3 NEG and KO cells were treated with miR‐29a, miR‐149‐3p and miR‐3934‐5p as well as miRctrl mimics and analyzed by western blot assay. The miR‐3934‐5p mimic significantly inhibited the EGFR signaling in PC3 cells with downregulated p1068‐EGFR and the phosphorylated p44/42‐MAPK (ERK1/2). (C) The normalized band densities of (B) were quantified with Student’s *t*‐test and are presented as mean ± SD (*n* = 3 individual experiments). *P < 0.05; **P < 0.01; ***P < 0.001. (D) The predicated interaction between miR‐3934‐5p and the EGFR 3’‐untranslated region (3’‐UTR) in TargetScan database. (E) PC3 NEG and KO cells were treated with EGFR inhibitor gefitinib and miR‐3934‐5p mimic, as well as DMSO as negative control and then examined for the migration through Transwell inserts, which were coated with either fibronectin (10 μg·mL^–1^) or laminin 111 (10 μg·mL^–1^). The cells that migrated through the insert pores and adhered to the bottom of the inserts were photographed. Scale bars: 200 µm. (F) Numbers of cells that migrated through the inserts were counted and compared between groups statistically using Student’s *t*‐test (mean ± SD, *n* = 3 independent experiments). *P < 0.05. (G) Effects of EGFR inhibitor gefitinib on the invasion of PC3 NEG and KO cells through Matrigel, in the presence of DMSO or Gefitinib (10 μΜ). The cells were pretreated with DMSO or gefitinib for 32 h prior to the experiments. The number of cells that invaded through the inserts was counted and compared between groups statistically using Student’s *t*‐test (mean ± SD, *n* = 3 independent experiments). *P < 0.05. (H) Effects of miR‐3935‐5p mimic on the invasion of PC3 NEG and KO cells through Matrigel, after the transfection of miRctrl or miR‐3935‐5p mimic. The number of cells that invaded through the inserts was counted and compared between groups statistically using Student’s *t*‐test (mean ± SD, *n* = 3 independent experiments). *P < 0.05.

Among all the 15 miRNA with EGFR as predicted target gene, we selected miR‐29a, miR‐149‐3p and miR‐3934‐5p for further investigation. The RT‐PCR analysis of exosomal miRNA expression indicated that EWI‐2 knockout in PC3 cells significantly decreased exosomal levels of these three miRNA (Fig. 3A), which implies that EWI‐2 plays a regulatory role in exosomal miRNA levels.

### 3.3 Exosomal miR‐3934‐5p targets EGFR and inhibits prostate cancer cell movement of certain modalities

Because all three miRNA were regulated by EWI‐2 knockout in PC3 cells, we subsequently identified miRNA that effectively targeted EGFR and regulated EGFR signaling. Based on previous studies by other groups and our miRNA profile analysis, we chose three miRNA that were downregulated via EWI‐2 knockout for further investigation: (1) miR‐29a‐5p, which was reduced in prostate cancer tissues, based on a previous study [33]; (2) miR‐149‐3p, which was reported possibly to negatively regulate the MAPK pathway [34]; and (3) the poorly studied miR‐3934‐5p, which was largely downregulated in the exosomes of PC3 cells through EWI‐2 knockout based on miRNA profile analysis.

PC3 NEG and KO cells were then transfected with miR‐29a, miR‐149‐3p and miR‐3934‐5p mimics and analyzed by western blot assay. Interestingly, all of the enforced miRNA expression was able to inhibit phosphorylation of EGFR (Fig. 3B,C). On the basis of the best effect of the inhibition of EGFR expression and phosphorylation, we finally selected miR‐3934‐5p for our further investigation. The miR‐3934‐5p mimic successfully inhibited the motility of PC3 cells, which confirmed our hypothesis that miR‐3934‐5p is an effective miRNA that successfully inhibits EGFR activity (Fig. 3E‐H). To further confirm the effect of miR‐3934‐5p on EGFR signaling, p44/42‐MAPK (ERK1/2) and phosphorylated p44/42‐MAPK (ERK1/2) were also analyzed by western blot assay, and the transfection of the miR‐3934‐5p mimic indeed attenuated EGFR‐MAPK‐ERK signaling by downregulating the phosphorylation of EGFR and p44/42‐MAPK (ERK1/2) (Fig. 3B,C). To verify further that EGFR is the downstream target of miR‐3934‐5p, we performed bioinformatics analysis using the miRNA database TargetScan; the predicated interaction between miR‐3934‐5p and EGFR 3’‐untranslated region (3’‐UTR) is shown in Fig. 3D.

To examine whether miR‐3934‐5p could alter the prostate cancer cell activity by targeting EGFR, gefitinib, an EGFR inhibitor, was added to PC3 NEG and KO cells with DMSO as negative control. MiR‐3934‐5p mimic was also transfected to the PC3 cells. Because EWI‐2 alters the cell surface levels of adhesion proteins and tetraspanins (CD81, CD9, αV integrin), as analyzed by flow cytometry (Fig. 1D), and the EWI‐2 expression is frequently lost in invasive and metastatic cancer cells according to the previous study, we analyzed cell migration and invasion in PC3 NEG and CRISPR/Cas9 KO cells. Directional solitary cell migration onto fibronectin (FN) or laminin‐111 (LN) became markedly increased upon EWI‐2 knockout (Fig. 3E,F). The invasiveness through Matrigel of EWI‐2 knockout cells was also significantly increased compared with controls (Fig. 3G,H). Notably, the EGFR inhibitor gefitinib reduced the elevated migration on FN and LN111 of EWI‐2 knockout PC3 cells to levels comparable with those of the control cells (Fig. 3E,F), and Matrigel increased invasion (Fig. 3G), strongly suggesting that elevated EGFR signaling contributed to the increased migration and invasiveness of EWI‐2‐knockout cells. Similar effects were observed in gefitinib treatment and the miR‐3934‐5p mimic‐transfected cells, which indicated that the cell invasion was attenuated with the miRNA transfection. Both cell migration and invasion in the PC3 KO group were inhibited by the treatments, indicating that miR‐3934‐5p could counteract the efficacy of EGFR‐induced higher cancer cell motility (Fig. 3E‐H). These findings indicate that EWI‐2 inhibits cell invasion by Matrigel and directional solitary cell migration onto various ECM, possibly by regulating the sorting of miR‐3934‐5p into exosomes and further affecting the EGFR signaling.

### 3.4 EWI‐2 restrains prostate cancer cell motility by regulating exosomal miR‐3934‐5p enrichment and further confining EGFR‐MAPK‐ERK signaling

After identifying EGFR as the target of exosomal miR‐3934‐5p and clarifying the regulating role of EWI‐2 on the sorting and enrichment of miR‐3934‐5p into the exosomes, we then analyzed the functional roles of EWI‐2 in the regulation of prostate cancer cell signal transduction. First, EWI‐2 siRNA was generated for the preliminary analysis of the relative signaling pathway (Fig. S2A–C). As indicated in western blot analysis, upon EWI‐2 silencing, the Tyr^1068^‐phosphorylated EGFR was upregulated with higher AKT phosphorylation at Ser473 (Fig. S2F,G). Cell adhesion‐associated proteins were also analyzed; the result showed that the N‐cadherin was elevated upon EWI‐2 knockdown with no change in the E‐cadherin level (Fig. S2F,G). Interestingly, the cell–cell adhesion analysis indicated that EWI‐2 was able to maintain adhesion in both calcium‐dependent and calcium‐independent conditions (Fig. S2D,E). The EGFR inhibitor gefitinib was also used to rescue the phenotype. With gefitinib treatment as well as the DMSO as negative control, the excessive activation of phosphorylated EGFR and AKT as well as the higher N‐cadherin expression upon EWI‐2 knockdown was attenuated, based on the EGFR inhibitor treatment (Fig. S2F,G). Apparently, the results confirm that EWI‐2 indeed contributes to the suppressed activation of EGFR phosphorylation and suggest that the EWI‐2 is likely required for the suppression of EGFR‐mediated cancer cell motility.

**Fig. 4:**
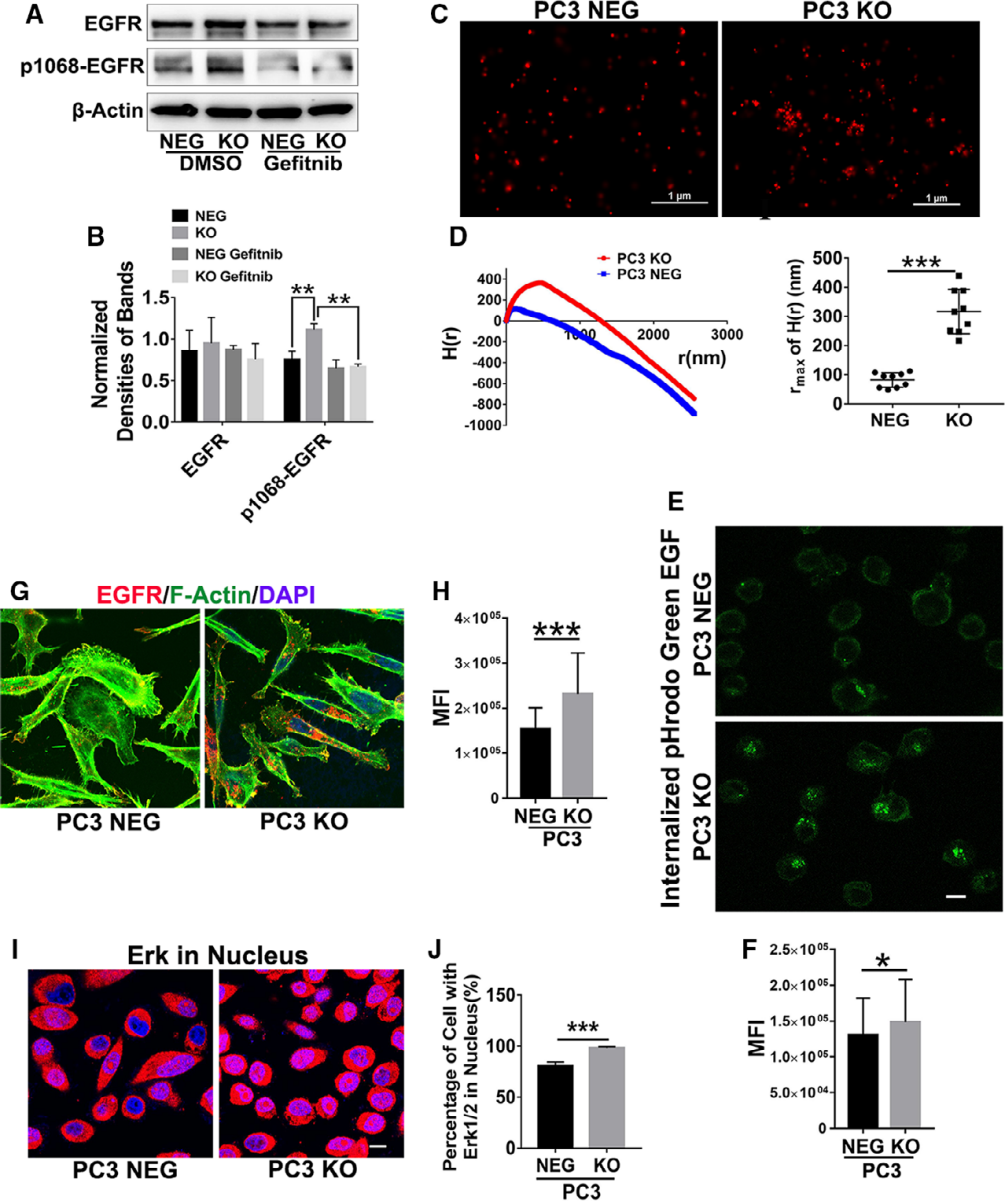
EWI‐2 removal potentiates EGFR‐MAPK signaling. (A) The EGFR activity was analyzed with Western blot in PC3 cells, together with actin as a loading control of whole‐cell lysates. (B) The normalized band densities were quantified with Student’s *t*‐test and presented as mean ± SD (*n* = 3 individual experiments). **P < 0.01. (C) EWI‐2 knockout resulted in higher EGFR clustering at the cell surface in PC3 cells. The cells were treated with EGF (25 nm) for 2 min, fixed, and stained with Alexa 647‐conjugated EGFR antibody. The EGFR distribution on the cell surface was imaged at nano‐scale by N‐STORM super‐resolution microscopy. Scale bars: 1 µm. (D) Ripley’s H functions were acquired by nis‐elements AR software (left), and the *r*_max_ of *H*(*r*) between groups was compared via independent samples *t*‐test (right) (mean ± SD, *n* = 3 independent experiments). ***P < 0.001. (E) EGF‐based endocytosis of EGFR was analyzed with 2 μg·mL^–1^ pHrodo green EGF conjugate in LCIS with incubation for 15–20 min at 37 °C. Cells were then washed in LCIS and fixed. Cells were imaged under N‐STORM imaging modality (Nikon) confocal microscope. (F) The mean fluorescence intensity of internalized green EGF was analyzed with imagej software and compared between groups statistically using Student’s *t*‐test (mean ± SD, *n* = 3 independent experiments). *P < 0.05. (G) Antibody‐based endocytosis of EGFR. The cells were incubated with EGFR mAb (1 μg·mL^–1^) and then incubated at 37 °C for 15–20 min, followed by three washes of 0.1 m glycine/1.0 m NaCl solution (pH 2.5). The cells were then fixed, permeabilized, incubated with secondary antibody and imaged with confocal microscopy. Scale bar: 10 µm. (H) The mean fluorescence intensity of internalized EGFR was analyzed with imagej software and compared between groups statistically using Student’s *t*‐test (mean ± SD, *n* = 3 independent experiments). ***P < 0.001. (I) The cells were fixed, permeabilized, incubated with ERK1/2 antibody and then fluorochrome‐conjugated 2nd antibody, and imaged with confocal microscopy. Scale bar: 10 µm. (J) The percentage of cells with nuclear ERK1/2 staining was quantified using Student’s *t*‐test and presented as mean ± SD (*n* = 3 individual experiments). ***P < 0.001.

Based on our results with EWI‐2 knockdown, we focused on the investigation of the role of EGFR‐ERK signaling in EWI‐2 function, as EGFR and TEM crosstalk [15] and serve as the target of miR‐3934‐5p, which is regulated by EWI‐2 as described above. After EWI‐2 knockout in PC3 cells, EGFR‐ERK signaling was markedly upregulated, with more Tyr^1068^‐phosphorylated EGFR (Fig. 4A,B), and increased ERK phosphorylation or activation (Fig. 3B,C). These changes indicate elevated EGFR signaling upon EWI‐2 knockout. Furthermore, nuclear translocation of ERK1/2 was obviously enhanced upon EWI‐2 knockout in PC3 cells (Fig. 4I,J). In addition to the EGFR signaling change, there were also changes in several cancer‐associated proteins, such as vimentin; the marker of epithelial‐to‐mesenchymal transition (EMT) was upregulated in PC3 knockdown cells and the EMT regulator β‐catenin was upregulated upon EWI‐2 knockdown. Conversely, the tumor suppressor protein Merlin was downregulated (Fig. S2H,I). Collectively, these results provide the basis for the theoretical research on whether and how EWI‐2 regulates prostate cancer motility.

To determine how EWI‐2 restrains EGFR activation, we analyzed the EGFR distribution in PC3 cells by super‐resolution imaging. As expected, clustering of EGFR at the cell surface was markedly increased in EWI‐2 knockout cells (Fig. 4C). Right shifts of the peaks of Ripley’s H function underline larger sizes in EGFR clusters in the EWI‐2 knockout cells (Fig. 4D). Together, these findings support an inverse correlation between EWI‐2 expression and EGFR‐MAPK‐ERK signaling. In addition to the membrane distribution of EGFR, EGFR internalization experiments were performed to evaluate the transduction of signaling molecules. In our study, we previously demonstrated that Cav‐1 and PTRF were all upregulated upon EWI‐2 knockout (Fig. 1H). Combined with the sustained EGFR activation and cell surface clustering caused by the elevated internalization and recycling, these results indicated that the clathrin‐independent internalization might also be elevated. Functionally, in the EGF‐stimulated EGFR internalization analysis, the depletion of EWI‐2 in PC3 cells upregulated the internalized EGFR (Fig. 4E,F). In addition, anti‐EGFR antibody‐based endocytosis was also elevated (Fig. 4G,H). It was previously demonstrated that at higher concentrations of ligand, the EGF receptor is also endocytosed through a clathrin‐independent, lipid raft‐dependent route, especially caveolae [35]. These data imply that EWI‐2 is important for the regulation of the internalization of EGFR, and during the high level of EGFR activation caused by the downregulation of miR‐3934‐5p upon EWI‐2 knockout, the lipid raft‐dependent pathway also plays a role in the mutual balance with classical clathrin‐independent endocytosis.

### 3.5 The expression and location of EWI‐2 in prostate cancer cells, *in vivo and in vitro*

**Fig. 5:**
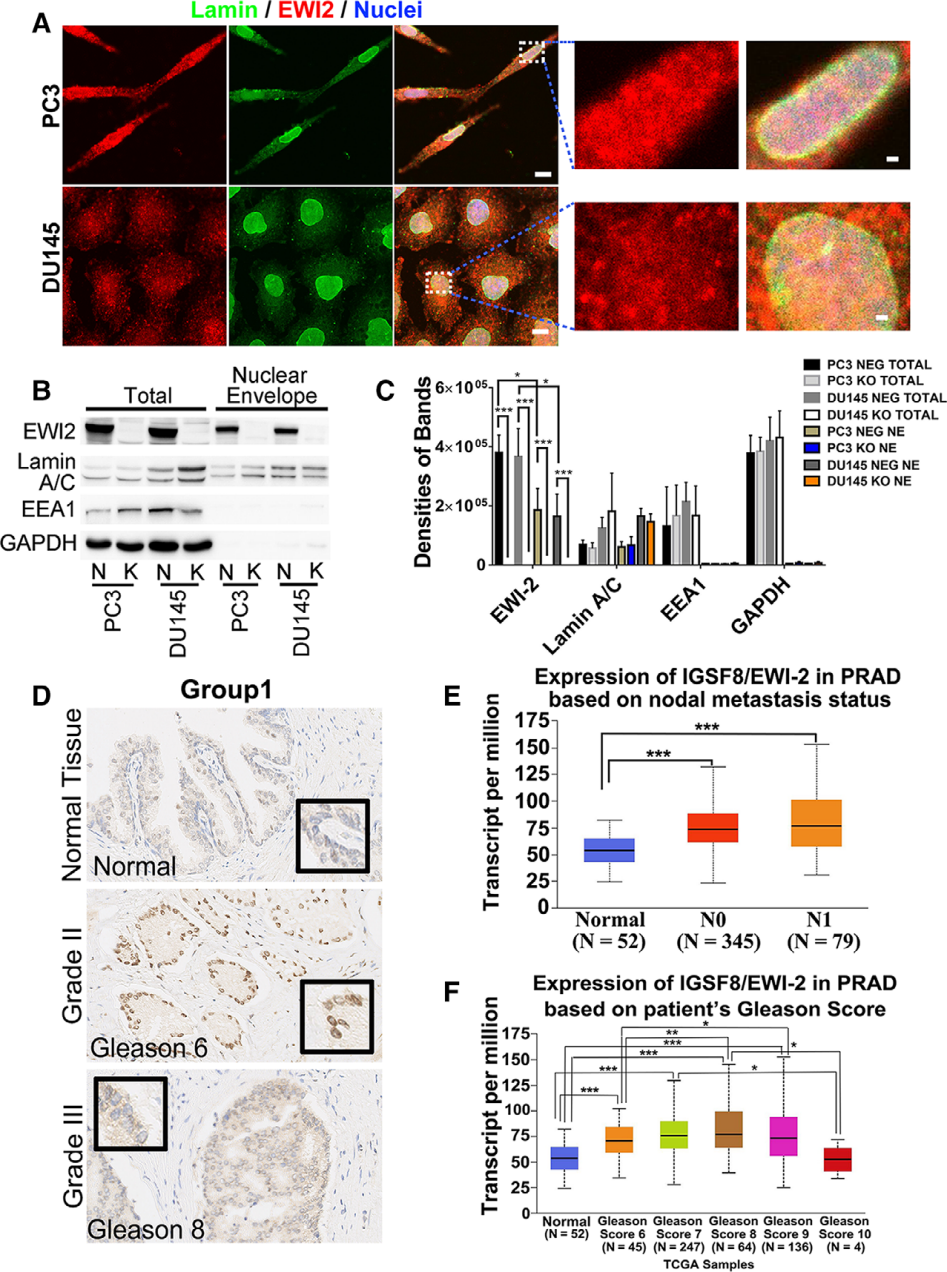
EWI‐2 localizes on the nuclear envelope. (A) PC3 and Du145 cells were labeled with Alexa 488‐conjugated lamin A/C mAb and EWI‐2 pAb, then Alexa 647‐conjugated secondary antibody. Images were captured with confocal fluorescence microscopy. Scale bars: 10 µm. Insets: nuclear colocalization of EWI‐2 with Lamin A/C in PC3 and Du145 cells. Scale bars: 1 µm. N, NEG; K, KO. (B) Total and NE proteins were extracted from the PC3 and DU145 cells and examined for EWI‐2 and other indicated proteins in western blot assay. Lamin A/C was used as the marker of NE and EEA1 served as the marker of cytoplasm. (C) The band densities of (B) were quantified using Student’s *t*‐test and are presented as mean ± SD (*n* = 3 individual experiments). *P < 0.05; ***P < 0.001. (D) Immunohistochemistry analysis on EWI‐2 proteins in human normal prostate and prostate cancer tissues. The tissues were co‐stained with hematoxylin for nuclei. Scale bar: 50 µm. (E,F) Expression of EWI‐2/IGSF8 in prostate adenocarcinoma based on metastasis status (D) and Gleason score (E) in TCGA database (http://ualcan.path.uab.edu/cgi‐bin/ualcan‐res.pl). The expression level of EWI‐2/IgSF8 between groups was compared via Student’s *t*‐test for multiple groups and the error bar was presented with the confidence interval (CI). The precise *P*‐values of (D,E) are presented in Supporting Information Fig. S4. *P < 0.05; **P < 0.01; ***P < 0.001.

EWI‐2 is mainly localized on the cell membrane and plays function roles in the cell adhesion and regulating the cancer cell motility. In our previous research, we confirmed the previous report about the colocalization of EWI‐2 with ERM protein [29] on the cell membrane with proximity ligation assay (PLA) (data not shown). Now, we also demonstrated that as a transmembrane protein, EWI‐2 is able to regulate miRNA sorting into exosomes. Interestingly, we determined the location of EWI‐2 on the NE of the PC3 and Du145 prostate cancer cell lines. According to the confocal assay, EWI‐2 colocalized with Lamin protein, which serves as the marker of NE in both cell lines. Clear colocalization of EWI‐2/Lamin was observed in the perinuclear region (Fig. 5A). To further confirm the expression of EWI‐2 on the nuclear envelope, nuclear envelope proteins were extracted and analyzed by western blot assay (Fig. 5B,C). These data indicate that in addition to its functional role on the cell membrane, EWI‐2 is also highly expressed on the NE and possibly shuttles nucleic acids and proteins in and out of the nucleus, which supports the conceptions and hypotheses that EWI‐2 regulates the sorting of miRNA into exosomes.

Based on the observation, EWI‐2 may be a protein which prefers to associate with lipid molecular layer structures. To confirm this hypothesis, a human tissue microarray was used. Upon IHC analysis, we found that the perinuclear staining expression of EWI‐2 protein was relatively low in normal prostate tissues but was increased in Grade II prostate cancer. However, staining signals decreased to a lesser extent or stopped, with no further rise in human metastatic prostate cancers compared with Grade II prostate cancer (Fig. 5D, Supporting Information Fig. S3), consistent with the EWI‐2 expression level in TCGA results (Fig. 5E,F). In Grade III prostate cancer tissues, EWI‐2 expression in some samples (e.g. group 2) resembled that in Grade II prostate cancer, whereas in other patients (e.g. groups 1 and 3), it resembled that in normal prostate tissues. Moreover, according to the TCGA results regarding EWI‐2 expression in prostate cancer patients, the expression level of EWI‐2 was relatively higher in patients with nodal metastasis (in comparison with normal prostate tissue). However, there was no more upregulation in N1 patients than in N0 patients (Fig. 5E, Supporting Information Fig. S4A). Nevertheless, an obvious downregulation of EWI‐2 expression in PRAD patients with a Gleason score of 10 was observed compared with the patients with a Gleason score of 6–9, resembling that in the patients with normal prostate tissue. These findings indicate that the change in EWI‐2 protein level is associated with the progression of prostate cancer; moreover, the perinuclear distribution of EWI‐2 indeed exists in prostate cancer cells and tissues and may serve as a regulator of the translocation shuttling molecule on both the cell membrane and NE.

### 3.6 EWI‐2 inhibits prostate cancer metastasis but not tumor growth

**Fig. 6:**
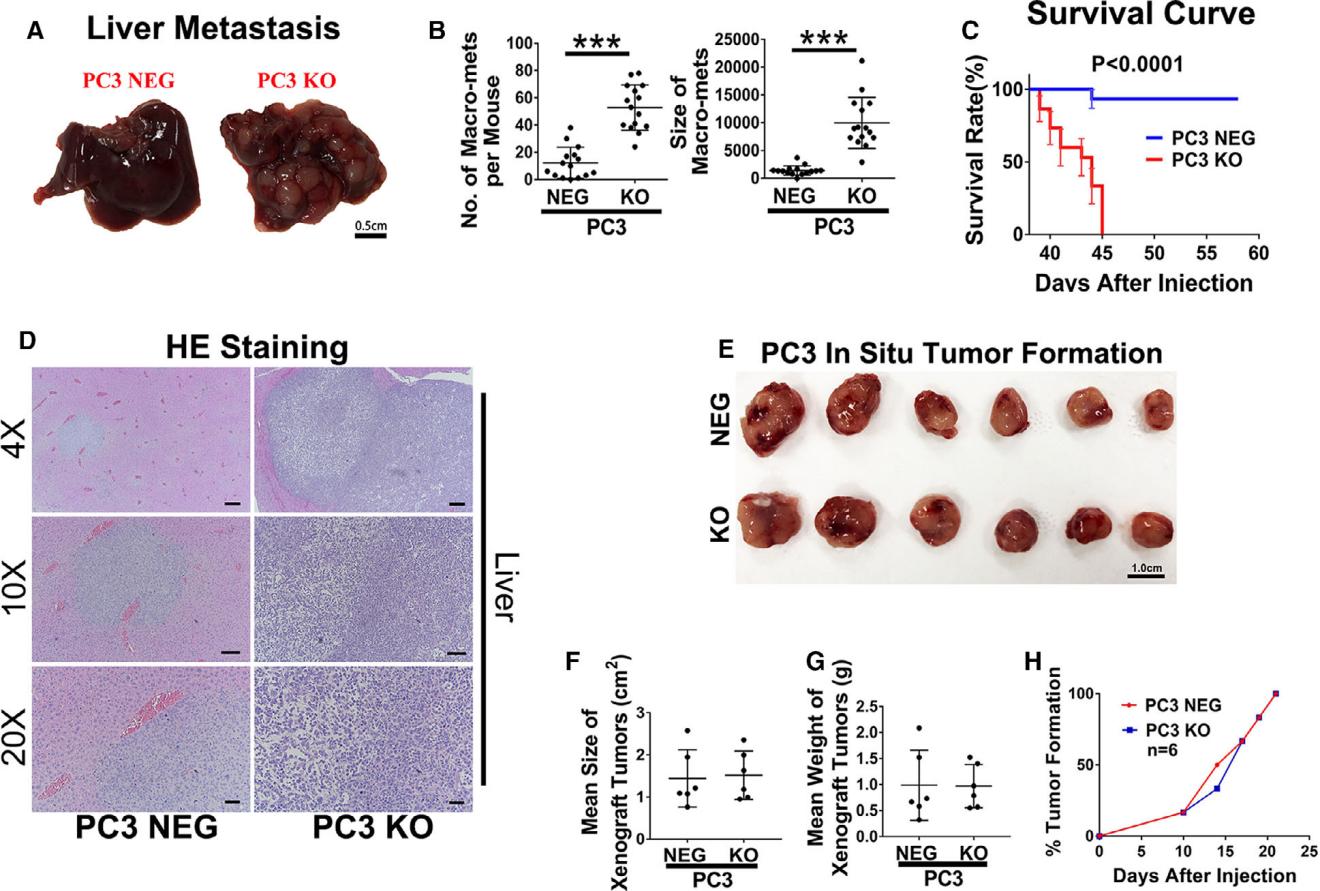
EWI‐2 removals promote tumor cell liver metastasis. (A,B) Liver metastasis of PC3 cells is elevated upon EWI‐2 knockout, in experimental metastasis assay. Two million PC3‐EWI‐2 knockout or control cells per mouse were administered through tail veins of NOG mice. After 45 days, the KO mice were euthanized, and the livers were dissected and imaged. The NEG mice were euthanized by day 58. Tumor metastatic lesions that appeared on the surface of livers were counted and measured. The data were quantified using Student’s *t*‐test and are presented as the number and size of macro‐mets per liver (mean ± SD, *n* = 3 individual experiments, 15 mice per group). ***P < 0.001. (C) Comparison of overall survival in Kaplan–Meier curve in the experimental metastasis assay with PC3 cells. Log rank test was used for the quantification (mean ± SD, *n* = 3 individual experiments, 15 mice per group). ***P < 0.001. (D) HE staining and images of the mouse liver sections. Scale bars: 4×, 250 µm; 10×, 125 µm; 20×, 50 µm. (E) EWI‐2 does not inhibit primary tumor xenograft growth. PC3 cells (4.0 × 10^6^ cells/administration) of NOD SCID mice were injected subcutaneously. The tumors were collected and weighed, and the sizes measured and then quantified with graphpad prism software. No significant difference was detected either in size (F) or weight (G) of the tumors. The tumor growth rate was recorded at days 0, 10, 14, 17, 19 and 21 until all mice had a tumor. The data were quantified using Student’s *t*‐test (F,G) and log rank test (H); no significant difference was observed between two groups (H) (*n* = 6 individual experiments, 18 mice per group).

To investigate the effect of EWI‐2 on prostate cancer metastasis, immune‐deficient mice were administered intravenous (i.v.) injection of PC3 cells. Starting approximately from the 6^th^ week after tail vein injection, the mice injected with PC3‐EWI‐2 knockout cells became moribund or displayed significant loss of bodyweight and were then euthanized, whereas the mice with PC3 control cells behaved largely normally until the 8th week or the end of the experiments (Fig. 6C). The livers of the mice with PC3‐EWI‐2 knockout cells showed profound metastasis with many more and larger macro‐metastasis lesions than the control group (Fig. 6A,B,D). Neither macro‐ nor micrometastatic lesions were observed in lungs in either group (Supporting Information Fig. S5).

We then tested the tumorigenic effect of EWI‐2 knockout cells in NOD SCID mice, in which PC3 cells were subcutaneously (s.c.) injected. We also confirmed that the cell viability and apoptosis were unchanged in prostate cancer cells with the depletion of EWI‐2 (Figure S7, Appendix S1). At the injection sites of primary tumor growth in NOD SCID mice receiving control or EWI‐2 knockout cells, tumors exhibited relatively the same size (Fig. 6E,F) and weight (Fig. 6E, G), as well as amount of tumor growth(Fig. 6H) 21 days postinjection. We also used another prostate cell line to investigate further the role of EWI‐2 in the regulation of tumor growth (Fig. S6A‐D). In Du145 cells, the depletion of EWI‐2 did not promote tumor growth; but it did support tumor formation when compared with PC3 cells regarding the tumor growth rate (Fig. S6, Appendix S1). Thus, together with the elevated tumor metastasis with EWI‐2 knockout, it is very likely that EWI‐2 can regulate prostate cancer cell metastasis but not tumor growth via the suppression of migration and invasion.

## 4 Discussion

As the second most common cancer among men, prostate cancer (PCa) prevalence has recently increased globally [36]. Digital rectal examination and biological markers such as serum prostate‐specific antigen (PSA) are common diagnostic methods for prostate cancer. In addition, there are other prognostic biomarkers based on SNP panels or epigenetic modifications. However, the current tools are either invasive procedures or have a relatively high negative predictive value [37]. In recent years, the overall mortality rates of prostate cancer have actually declined, particularly in developed countries, due to successful diagnosis and treatment [38], which indicates the importance of early‐stage diagnosis of prostate cancer as well as the screening of PCa with high metastatic potential.

Recently, exosomes carrying miRNA have been a subject of great interest as potential biomarkers for liquid biopsy, such as serum, semen and urine samples of patients with PCa, which can overcome the limitations of previously used biomarkers such as PSA [20, 39, 40, 41, 42, 43, 44]. Thus, with the expectancy of identifying more effective biomarkers for prostate cancer detection, this study aimed at investigating the roles of EWI‐2 in the regulation of miRNA sorting to the exosomes, as well as the translocation of signaling molecules and at investigating further the effect of EWI‐2 on prostate cancer metastasis.

### 4.1 EWI‐2 inhibits Caveolin‐1 expression and further regulates the sorting of exosome

It has been proven that EWI‐2 is a specific exosome glycoprotein that serves a function at N‐linked glycan sites and directly impacts the recruitment of exosomes [45]. It is also known that nucleotides and proteins are selected specifically into EV cargo rather than randomly [46]. In our study, we demonstrated that EWI‐2 is highly expressed in exosomes extracted from prostate cancer cells, which indicates that EWI‐2 may functionally regulate the selection of EV cargo proteins and nucleotides.

Recent findings have demonstrated that as the major resident scaffolding protein required for caveolae formation that facilitates endocytosis and exocytosis of particles, Cav‐1 plays a crucial role in the biogenesis of exosomes [26, 47]. Cav‐1 is the first identified membranous protein that directly guides RNA‐binding protein into extracellular vesicles [47]. Interestingly, an increasing number of reports have demonstrated that Cav‐1 regulates tumor–host interactions by promoting tumor progression, growth, metastasis, therapy resistance and cell survival in different cancer types such as breast cancer [48], lung cancer [49], bladder cancer [50], and prostate cancer [51]. Considering the essential role of Cav‐1 in the cancer metastasis, we think that the upregulated prostate cancer cell metastasis upon EWI‐2 knockout may be correlated with these protein changes. More interestingly, the emerging of caveolae formation in PC3 cells upon EWI‐2 knockout is a positive regulator of the membrane curvature formation that facilitates the exosome biogenesis or packaging.

Because we elucidated the role of EWI‐2 in the biogenesis and secretion of exosomes, even though the detailed mechanisms remain unclear, we note that the EWI‐2 knockout restored PTRF expression in PC3 cells. It should be emphasized that PC3 cells express abundant Cav‐1 but not PTRF, and the ectopic expression of PTRF in PC3 cells is needed for caveolae formation. However, it was reported that the absence of PTRF in PC3 cells notably contributes to tumor metastasis [52]. There is a contradiction here with the restored PTRF with the elevated prostate cancer cell motility in our results. We herein explain this point using the following aspects:

- The emerging of PTRF expression may be due to the lack of EWI‐2 expression in PC3 KO cells, which may be a membranous key regulator of the exosome formation. The absence of PTRF in PC3 cells leads to the formation of noncaveolar Cav‐1 lipid rafts [53]. Cav‐1 is the major structural component of caveolae and the restored expression of PTRF in PC3 cells sequesters Cav‐1 into caveolae and then further participates in a range of cellular processes such like extracellular vesicle formation and endocytosis [53]. Interestingly, the role of PTRF in the smallest extravcellular vesicles, the so‐called exosome, is not yet clear [46]. So the emerging PTRF here is basically working impacting endocytosis due to the activation of EGFR.
- EGFR was activated due to EWI‐2 knockout. It was reported that PTRF/cavin‐1 overexpression increased exosome secretion both *in vitro* and *in vivo*, and is regulated in the EGFR/PI3K/AKT pathway [54]. This gives us new clues about the role of PTRF restoration in PC3 cells that do not have a functional effect on the inhibition of prostate cancer metastasis or may be balanced by the EGFR activation.

### 4.2 The cell membrane and nuclear envelope location of EWI‐2 shuttle the translocation of signaling protein and microRNA

Although the role of PTRF in the biogenesis and packaging of exosome has been defined, more studies are needed to clarify how EWI‐2 is directly involved in exosome formation and the sorting of extracellular vesicle cargo into the exosome. It was reported that EWI‐2 cytoplasmic tail actively engages with the cell membrane via PIP binding and palmitoylation [29]. Interestingly, a recent discovery showed the location of phosphoinositides in the eukaryotic cell nuclear envelope [55], which indicated that EWI‐2 may exist with PIP not only on the cell membrane but also on the NE. These estimations are based on the assumption that EWI‐2 may serve as a gatekeeper on the nuclear envelope and control the translocation of signaling molecules such as miRNA and signaling proteins.

MicroRNA are enriched in both the cytoplasm and the nucleus [56]. It was reported that microRNA synthesis is a multistep process that requires the transportation of RNA from the nucleus to the cytoplasm through nuclear pore complexes via mobile export receptors [28]. Our findings provide evidence that in addition to the regulation of exosome packaging, the expression of EWI‐2 on the membrane and NE plays an important role in the miRNA sorting from the nucleus and cytoplasm to exosomes. A series of functional experiments revealed the important role of miRNA in prostate cancer tumorigenesis [57, 58, 59]. In the present study, a human miRNA microarray was used to analyze the exosomal miRNA expression profiles, and we obtained evidence that in addition to the role of PTRF/Cav‐1 in the sorting of extracellular vesicle cargo to exosomes, EWI‐2 also plays an important role via its specific location. The removal of EWI‐2 regulates several miRNA in exosomes, which functionally impact multiple signaling pathways.

The investigation of the exosomal miRNA profile provides us with important clues for further signaling studies. It has been reported that miR‐361‐3p was significantly downregulated in prostate secretion samples of PCa patients [59]. KEGG pathway analysis showed that the MAPK pathway may be negatively regulated by the corresponding miRNA such as miR‐149‐3p [34]. Konoshenko et al. reported that miR‐92a is a cell‐free miRNA that has diagnostic potential for prostate cancers [60]. Loss of miR‐378 in prostate cancer correlates with the prediction of short‐term relapse of prostate cancer patients [61]. Kato et al. reported that miR‐29a was markedly reduced in prostate cancer tissues [33]. MiR‐491‐5p was also reported to have a negative effect on the regulation of cell proliferation and motility by targeting PDGFRA in prostate cancer [62]. In addition, androgen downregulation of miR‐760 was able to regulate interleukin (IL)‐6 and then promote prostate cancer cell growth [63], and there was less expression of miR‐942 in high‐grade than in low‐grade prostate cancer cases at biopsy [64]. Together with the KEGG pathway analysis, we predict that the EGFR‐MAPK pathway is highly correlated with EWI‐2 and may be affected by the EWI‐2 knockout in the prostate cancer cells.

### 4.3 EWI‐2 restrains EGFR‐MAPK‐ERK signaling through regulating exosomal miR‐3934‐5p enrichment

A recent study has identified EGFR involvement in the suppression of tumor suppressor‐like miRNA maturation in response to hypoxic stress through phosphorylation of argonaute 2 (AGO2) [65]. According to the analysis of the exosomal miRNA profile and the EGFR signaling change based on EWI‐2 depletion, we demonstrate that exosomal miR‐3934‐5p, which was confirmed as an EGFR inhibitor, might be functionally delivered to parental prostate cancer cells, possibly in an autocrine/paracrine manner, and further affect the EGFR‐MAPK‐ERK signaling of other cancer cells. Basically, EWI‐2 depletion forces miR‐3934‐5p to be packaged into exosomes and transferred outside of cells, eventually acting as a bridge between different cancer cells and activating EGFR signaling.

Taking into account exosome formation as well as the sorting of miRNA to exosomes, we previously discussed the role of Cav‐1 in the biogenesis and packaging of exosomes. However, Cav‐1 may also engage in the internalization of EGFR. The endocytosis of EGFR is mainly internalized via clathrin and is essential for sustained EGFR signaling [66]. However, different ligands may trigger alternative routes of EGFR internalization, such as Cav‐1‐mediated endocytosis [67, 68]. In our study, the EGFR signaling and internalization study together with the expression of Cav‐1 change give us new clues as to the biological functions of how EWI‐2 regulates EGFR signaling and recycling within cells. It is interesting that the Cav‐1 change caused by EWI‐2 depletion plays two roles in this story:

- supporting caveolae formation and in turn regulating exosome formation as well as the sorting of miRNA in and out of the exosome;
- partially governing the internalization of EGFR within the cells, which may serve as an auxiliary function for clathrin‐dependent endocytosis or play a balance function in the process. Nevertheless, the mechanism by which EWI‐2 regulates caveolin‐dependent internalization needs further investigation as well as in‐depth study.

As demonstrated before, the transcriptional responses caused by ERK1/2 activation [69] and ERK translocation into the nucleus are needed to enhance cell motility [70]. How EWI‐2 restrains the translocation of signaling molecules through the nuclear envelope remains unclear. It is plausible to postulate that EWI‐2 acts on similar molecular events at or near both membranes, such as vesicular fission and fusion processes. Notably, EWI‐2 localization at peri‐nuclear region, presumably at or near the NE, was observed at the intermediate progression stage of prostate cancer, implying the pathological relevance of this subcellular distribution. These findings from our study strongly suggest that EWI‐2 hinders outside–in signaling at and between the plasma and NE. Together, besides the localization of EWI‐2 on cell membrane demonstrated before by other groups, these findings imply: (1) interaction of EWI‐2 with NE, (2) a regulatory role for EWI‐2 in shuttling cargos through nuclei and exosomes and (3) the ability of EWI‐2 to control EGFR‐MAPK signaling via the regulation of miR‐3934‐5p sorting to exosomes. Thus, understanding the molecular mechanisms of EWI‐2 in prostate cancer cells may contribute to the development of new effective examinations and treatment methods for this disease.

### 4.4 EWI‐2 serves as a negative regulator of prostate cancer metastasis but not tumor growth

Combining the expression level of EWI‐2 mRNA in prostate cancer from the TCGA database and the TMA data with the comparative *in vivo*/*in vitro* analyses allowed us to conclude that EWI‐2 inhibits metastasis of prostate cancer. Based on this, we investigated whether EWI‐2 leads to inhibition of prostate cancer cell metastasis. We found that knockout of EWI‐2 in PC3 cells indeed elevates liver metastasis in an immune‐deficient mouse model with intravenous injection. This is consistent with previous observations on EWI‐2 in other cancer types [11, 71]. It should be emphasized that EWI‐2 depletion leads to liver metastasis but not lung metastasis, which may result from elevated integrin αV expression in PC3 EWI‐2 KO cells. According to a previous study, Integrin αvβ3 plays a key role in liver metastasis [72]. Another study also reported that the exosomal integrin could regulate local microenvironments within target metastatic organs, and integrin αv was linked to liver metastasis [73]. However, EWI‐2 expression in the exosomes and its regulation of integrin expression in exosomes remain to be established.

Previous reports have demonstrated that tumor‐derived exosomes contribute to tumor cell migration and tumor progression in an autocrine/paracrine manner [74, 75, 76]. It was also previously reported that exosomes could promote proliferation and metastasis of the parental cancer cells such as hepatocellular carcinoma (HCC) [77]. Another study also demonstrated that the MAPK signaling pathway is activated and closely associated with tumor invasion and the metastasis in a wide spectrum of human tumors [78, 79]. In our study, we found that EWI‐2 can regulate exosomal miRNA profiles and further demonstrated that changes in exosomal miRNA, such as miR‐3934‐5p, based on EWI‐2 knockout are highly correlated with MAPK signaling and cell adhesion signaling transduction. EGFR was shown to regulate the activation of integrins and cell adhesion molecules, further controlling the extracellular environment [32]. MAPK signaling was reported and well demonstrated as being active in both early and advanced stages of tumorigenesis; it promotes cancer cell proliferation, survival and metastasis. In MAPK signaling, EGFR frequently acts as an upstream regulator of RAS/RAF/MEK/extracellular signal‐regulated kinase [79]. After analyzing the expression of the EGFR‐MAPK signaling pathway, the results indicated that as the most likely targeted pathway of EWI‐2‐associated miRNA, EGFR and its downstream p44/42 MAPK are activated by EWI‐2 knockout in PCa cells. Here, we describe fully the key role of EWI‐2 in regulating prostate cancer cell metastasis, which was controlled by the exosomal miRNA and its targeted EGF receptor. However, for the tumor growth investigation, the depletion of EWI‐2 in prostate cancer cells does not support tumor growth, and even shows a trend of lowering the speed of growth of tumors, mainly due to the unchanged cell viability and apoptosis in prostate cancer cells with the depletion of EWI‐2. These results are more or less consistent with a previous study on melanoma [10]. However, the mechanism by which EWI‐2 modulates the response of the recipient organ to circulating exosomes, which can regulate the cancer cell metastasis, requires further investigation.

## 5 Conclusions

In conclusion, we have demonstrated here that as a PIP partner, in addition to the membrane location, EWI‐2 also exists on the NE and exosomes and governs the translocation of signaling molecules between nucleus and cytoplasm, as well as the sorting of miRNA from the cell to exosomes. In the present study, we discovered a reverse correlation of exosomal miR‐3934‐5p expression levels with EGFR signaling as well as prostate cancer cell metastasis, which was regulated by EWI‐2. All the important changes lead to the regulation of EGFR‐MAPK‐ERK signaling and ultimately affect the role of EWI‐2 in the regulation or inhibition of prostate cancer metastasis. Together, these findings establish a novel role of EWI‐2 in the prostate cancer cell metastasis which provides us insights into the investigation of new detection or treatment methods for prostate cancers.

## Conflict of interest

We declare that we have no financial or personal relationships with other people or organizations that might inappropriately influence our work; there is no professional or other personal interest of any nature or kind in any product, service and/or company that could be construed as influencing the position presented in, or the review of, the manuscript entitled: ‘*EWI‐2 controls nucleocytoplasmic shuttling of EGFR signaling molecules and miRNA sorting in exosomes to inhibit prostate cancer cell metastasis’*. by Chenying Fu et al.

## Author contributions

CF and QW conceived and designed the project. CF, QZ and SY worked on the experimental studies. CF and QZ worked on the data acquisition, analyzed and interpreted the data. AW and CF were responsible for statistical analysis. CF and QW wrote the paper and edited the manuscript. YJ was responsible for manuscript revision/review. LB worked on the STORM experiments. QW approved the final version of the manuscript.

### Peer Review

The peer review history for this article is available at https://publons.com/publon/10.1002/1878‐0261.12930.

## Supporting information


**Fig. S1.** Nanoparticle tracking analysis of PC3 cells.

**Fig. S2.** EWI‐2 silencing in PC3 cells regulates cell–cell adhesion and mutiple signaling.

**Fig. S3.** EWI‐2 expression and distribution in human prostate tissues.

**Fig. S4.** Statistical significance of the expression of EWI‐2/IGSF8 in TCGA database.

**Fig. S5.** EWI‐2 does not inhibit lung metastasis of PC3 prostate cancer cells.

**Fig. S6.** EWI‐2 does not inhibit primary tumor xenograft growth in Du145 cells.

**Fig. S7.** EWI‐2 does not alter the viability or apotosis of prostate cancer cells.

**Appendix S1.** Materials and methods.

## Data Availability

The data that support the findings of this study are available from the corresponding author upon reasonable request.
