# Supplementary material for: EWI‐2 controls nucleocytoplasmic shuttling of EGFR signaling molecules and miRNA sorting in exosomes to inhibit prostate cancer cell metastasis
Source: Mol Oncol. 2021 Mar 27;15(5):1543–65. doi: 10.1002/1878-0261.12930 (PMC8096798; doi:10.1002/1878-0261.12930)
Supplement: Supplementary file 1 — Fig. S1. Nanoparticle tracking analysis of PC3 cells. [file MOL2-15-1543-s007.pdf]

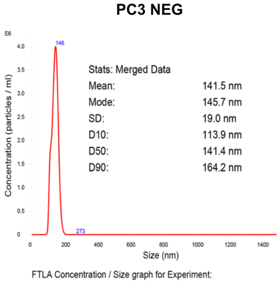
PC3 NEG
E6
146
273
Stats: Merged Data
Mean: 141.5 nm
Mode: 145.7 nm
SD: 19.0 nm
D10: 113.9 nm
D50: 141.4 nm
D90: 164.2 nm
Concentration (particles / ml)
Size (nm)
FTLA Concentration / Size graph for Experiment:

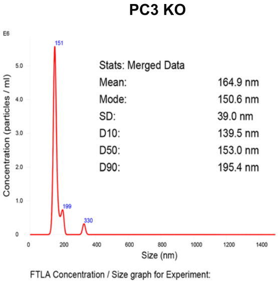
PC3 KO
E6
151
199
330
Stats: Merged Data
Mean: 164.9 nm
Mode: 150.6 nm
SD: 39.0 nm
D10: 139.5 nm
D50: 153.0 nm
D90: 195.4 nm
Concentration (particles / ml)
Size (nm)
FTLA Concentration / Size graph for Experiment:

Figure S1
