# Supplementary figures and images for: EWI‐2 controls nucleocytoplasmic shuttling of EGFR signaling molecules and miRNA sorting in exosomes to inhibit prostate cancer cell metastasis
Source: Mol Oncol. 2021 Mar 27;15(5):1543–65. doi: 10.1002/1878-0261.12930 (PMC8096798; doi:10.1002/1878-0261.12930)

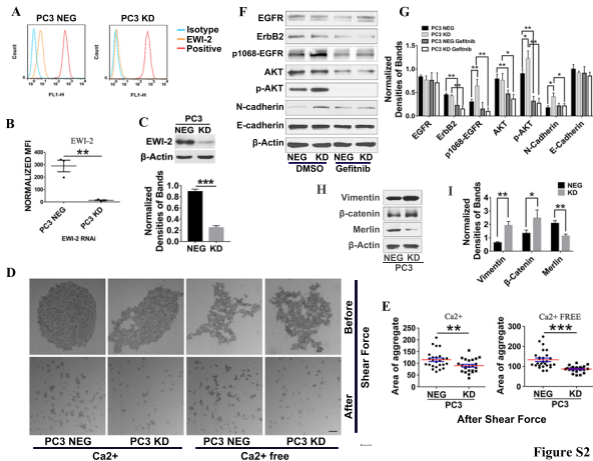

Figure S2

Supplement: Supplementary file 2 — Fig. S2. EWI‐2 silencing in PC3 cells regulates cell–cell adhesion and mutiple signaling. [file MOL2-15-1543-s002.pdf]

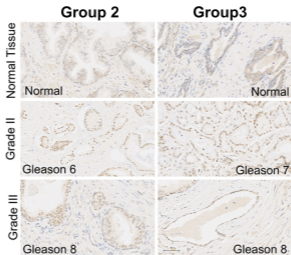

**Figure S3**

Supplement: Supplementary file 3 — Fig. S3. EWI‐2 expression and distribution in human prostate tissues. [file MOL2-15-1543-s001.pdf]

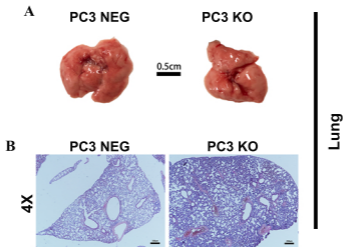

**Figure S5**

Supplement: Supplementary file 5 — Fig. S5. EWI‐2 does not inhibit lung metastasis of PC3 prostate cancer cells. [file MOL2-15-1543-s003.pdf]

A

## Du145 In Situ Tumor Formation

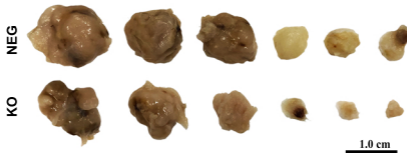

B

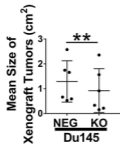

C

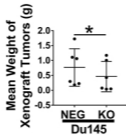

D

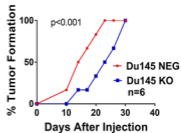

Figure S6

Supplement: Supplementary file 6 — Fig. S6. EWI‐2 does not inhibit primary tumor xenograft growth in Du145 cells. [file MOL2-15-1543-s005.pdf]

**A**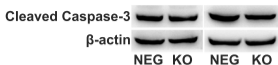**B**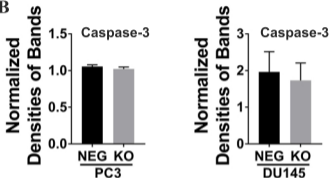**C**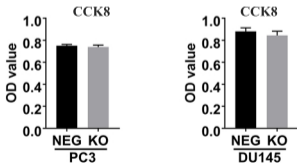**Figure S7**

Supplement: Supplementary file 7 — Fig. S7. EWI‐2 does not alter the viability or apotosis of prostate cancer cells. [file MOL2-15-1543-s004.pdf]
