# Supplementary material for: EWI‐2 controls nucleocytoplasmic shuttling of EGFR signaling molecules and miRNA sorting in exosomes to inhibit prostate cancer cell metastasis
Source: Mol Oncol. 2021 Mar 27;15(5):1543–65. doi: 10.1002/1878-0261.12930 (PMC8096798; doi:10.1002/1878-0261.12930)
Supplement: Supplementary file 4 — Fig. S4. Statistical significance of the expression of EWI‐2/IGSF8 in TCGA database. [file MOL2-15-1543-s006.pdf]

A

N0	No regional lymph node metastasis	N1	Metastases in 1 to 3 axillary lymph nodes
N2	Metastases in 4 to 9 axillary lymph nodes	N3	Metastases in 10 or more axillary lymph nodes

Comparison	Statistical significance	
Normal-vs-N0	2.22044604925031E-16	***
Normal-vs-N1	1.67500013859012E-10	***
N0-vs-N1	1.444440E-01	

*p<0.05 **p<0.01 ***p<0.001

B

Comparison	Statistical significance	
Normal-vs-Gleason score 6	4.04669999953811E-07	***
Normal-vs-Gleason score 7	1.11022302462516E-16	***
Normal-vs-Gleason score 8	1.38159483853428E-11	***
Normal-vs-Gleason score 9	3.78252984489791E-13	***
Normal-vs-Gleason score 10	8.693600E-01	
Gleason score 6-vs-Gleason score 7	9.517800E-02	
Gleason score 6-vs-Gleason score 8	5.038000E-03	**
Gleason score 6-vs-Gleason score 9	2.970300E-02	*
Gleason score 6-vs-Gleason score 10	5.464000E-02	
Gleason score 7-vs-Gleason score 8	5.429900E-02	
Gleason score 7-vs-Gleason score 9	3.125200E-01	
Gleason score 7-vs-Gleason score 10	1.880160E-02	*
Gleason score 8-vs-Gleason score 9	3.647200E-01	
Gleason score 8-vs-Gleason score 10	3.036400E-02	*
Gleason score 9-vs-Gleason score 10	9.184000E-02	

*p<0.05 **p<0.01 ***p<0.001

Figure S4
