# Supplementary material for: EWI‐2 controls nucleocytoplasmic shuttling of EGFR signaling molecules and miRNA sorting in exosomes to inhibit prostate cancer cell metastasis
Source: Mol Oncol. 2021 Mar 27;15(5):1543–65. doi: 10.1002/1878-0261.12930 (PMC8096798; doi:10.1002/1878-0261.12930)
Supplement: Supplementary file 8 — Appendix S1. Materials and methods. [file MOL2-15-1543-s008.docx]

**Supplementary Materials and Methods**

**Nanoparticle tracking analysis**

The extracellular vesicles were extracted as described previously and examined with NanoSight NS300 instrument (Malvern Instruments, Malvern, UK), by following the instruction provided by the manufacturer. The number and size of exosomes were analysed with the nanoparticle tracking analysis system (NTA 3.2 Dev Build 3.2.16) of the instrument.

**Cell-cell adhesion assays**

Cell-cell adhesion was examined by hanging-drop aggregation assay. Cells in suspension were prepared as hanging drops of calcium-containing or calcium-free media, and were incubated at 37^o^C overnight. Then, the cells went through shear stress and were photographed using light microscopy. Aggregates were quantified using Image J software. The results are displayed as the area of aggregate.

**Cell viability assay and apoptosis assay**

Cell Counting Kit-8 (CCK-8) assay kit was used for Cell viability assay in PC3 and DU145 cells. PC3 and DU145 cells (NEG and KO) were seeded in 96-well plates and cultured until ~90% confluence. The cell culture medium was then replaced with CCK-8 working solution and following by 1-hour incubation in 37℃ incubator. The absorbance of 450nm was then measured and quantified with paired Student’s *t*-test.

Cleaved caspase-3 was used to evaluate apoptosis of PC3 and Du145 cells with/without EWI-2 knockout by western blotting.

**Subcutaneous tumor formation assay**

Du145 NEG and KO cells (4.0 × 10^6^ cells/administration) were injected subcutaneously of NOD SCID mice (NOD.CB17-Prkdc^scid^/NcrCrl), which were obtained originally from Charles River Laboratories. The housing and handling conditions of the mice were consistent with the method above. The experiments were performed in accordance with the experimental animal ethics committee of West China Hospital, Sichuan University. The licence number approved for the experiments was 2020038A. The mice were weighed every 3 days after the injection and euthanized when tumor size reached 1.0`1.5cm. The tumors were collected and weighed, the sizes were measured, and then quantified with GraphPad Prism software.

**Statistical analyses**

Quantifications were typically based on three to four individual experiments. Kaplan-Meier survival curve was plotted and analyzed with the Log-rank (Mantel-Cox) test. Data are presented as the mean ± SD or standard error of the mean (SEM). Analyses were conducted with SPSS 13.0 software and GraphPad Prism 5.0. Student’s t-test was used to determine significance of difference, *, ** and *** represent *p* values less than 0.05, 0.01 and 0.001, respectively.
